# Microbiota-targeted interventions in chronic kidney disease: probiotics, prebiotics, synbiotics, fecal microbiota transplantation, and traditional Chinese medicine

**DOI:** 10.3389/fcimb.2026.1900027

**Published:** 2026-10-13

**Authors:** Liu-Cheng Li, Wen-Hui Cui, Zhi-Hui Zhang, Jing Song, Yao-Yao Xu, Ding-Ge Cai, Yan Guan, Kai-Li Mao

**Affiliations:** 1 Department of Pharmacy, Sir Run Run Shaw Hospital, Zhejiang University School of Medicine, Hangzhou, China; 2 Pharmacy Department, Sinopharm Tongmei General Hospital, Datong, China; 3 Institute of Vascular Anomalies, Shanghai TCM-Integrated Hospital, Shanghai University of Traditional Chinese Medicine, Shanghai, China; 4 Department of Pharmacy, Kunshan Hospital of Integrated Traditional Chinese and Western Medicine, Suzhou, China; 5 Department of Pharmacy, The People’s Hospital of Wenzhou, Suzhou, China; 6 The Department of Pharmacy, The Second Affiliated Hospital of Yunnan University of Chinese Medicine, Kunming, China; 7 Department of Pharmacy, Quzhou People's Hospital; The Quzhou Affiliated Hospital, Wenzhou Medical University, Quzhou, China

**Keywords:** fecal microbiota transplantation, kidney disease, microbiota therapeutics, traditional Chinese medicine, uremic toxins

## Abstract

Chronic kidney disease (CKD) is tightly associated with gut dysbiosis that fuels uremic toxin buildup, systemic inflammation, and renal fibrosis, yet conventional CKD therapies offer limited disease-modifying choices. This narrative review integrates preclinical and clinical evidence (searched PubMed, Web of Science, Cochrane up to May 31, 2026) for gut-microbiota interventions, probiotics, prebiotics, synbiotics, fecal microbiota transplantation (FMT), and traditional Chinese medicine (TCM), and compares their efficacy via network meta-analyses stratified by CKD stages. All microecological agents remodel gut flora, boost short-chain fatty acid producers and strengthen intestinal barriers to cut protein-bound uremic toxins, but eGFR benefits vary by strain, formulation, and disease stage, with most robust effects in non-dialysis CKD (stages 3-4). Dialysis patients gain minimal renal improvements, only mild anti-inflammatory and gastrointestinal relief. Network meta-analyses indicate distinct therapeutic strengths of each regimen, yet indirect cross-study comparisons yield low-to-moderate evidence certainty without direct head-to-head trials. FMT and herbal TCM show promising renoprotective gut-kidney regulatory effects in animal research and small preliminary human cohorts, though large controlled human trials remain absent. Subtype-specific CKD responses (diabetic nephropathy, IgA nephropathy) cannot be universally extrapolated. Despite encouraging mechanistic and early clinical signals, standardized testing, comparative trials and strict safety monitoring for fragile CKD patients are required before routine guideline use of gut-targeted therapies.

## 1 Introduction

Chronic kidney disease (CKD) is a progressive disorder marked by persistent abnormalities in kidney structure or function (Nagano et al., 2025). The conventional biomarkers, notably the estimated glomerular filtration rate (eGFR) and the urine albumin-to-creatinine ratio, have traditionally been central to its diagnosis and management (McDonnell et al., 2025). CKD typically unfolds as a multisystemic disorder fraught with protean complications that inexorably progress to end-stage kidney disease (ESKD), rendering patients dependent on lifelong renal-replacement therapy. This relentless trajectory profoundly erodes the quality of life and imposes an escalating, often overwhelming psychosocial and economic burden on patients and their families (Zaimi and Grapsa, 2024; Oza-Gajera et al., 2023). CKD stands as a major global health concern. Since 1990, its age-standardized prevalence has seen a marked increase of 29.3%, a trend coupled with significant rises in both morbidity and mortality rates (Collaboration, 2020; Voroneanu et al., 2023). Forecasts suggest that by 2040, CKD will emerge as the world’s fifth most common cause of mortality (Kalantar-Zadeh et al., 2021). It is essential to establish the core molecular pathways that drive CKD progression and renal fibrosis, as these mechanisms form the pathological foundation upon which gut-derived insults converge and against which the therapeutic effects of microecological interventions are measured.

Regardless of underlying etiology, CKD progression converges on a final common pathway characterized by progressive renal fibrosis driven by interconnected molecular mechanisms: the TGF-β/Smad and Wnt/β-catenin pathways promote extracellular matrix deposition and epithelial-mesenchymal transition; NF-κB-mediated inflammation, oxidative stress (involving NADPH oxidase and Nrf2 dysregulation), and mitochondrial dysfunction create a self-perpetuating cycle of cellular injury; and cellular senescence further accelerates renal aging and fibrosis. These pathways constitute the primary therapeutic targets for interventions aiming to slow CKD progression and provide the mechanistic context for understanding how gut-derived uremic toxins and inflammatory mediators exacerbate renal injury through the gut-kidney axis (Abbad et al., 2025; Liu et al., 2025a).

Early-stage CKD can be slowed by a combination of lifestyle enhancements, targeted dietary modifications, and evidence-based medication (de Boer et al., 2022). As kidney disease progresses, patients eventually require renal replacement therapies, such as hemodialysis or peritoneal dialysis, which sustain life but do not restore normal kidney function. It is crucial to closely manage the metabolic quartet: hyperglycemia, hypertension, dyslipidemia, and hyperuricemia. Proactively addressing downstream issues such as renal anemia and electrolyte imbalances is also essential. Therefore, pharmacotherapeutic strategies encompass targeted anti-hyperglycemic, antihypertensive, lipid-lowering, and urate-lowering agents, supplemented by iron repletion, erythropoiesis-stimulating agents, and meticulous electrolyte rebalancing to mitigate the systemic burden (Elendu et al., 2023). As kidney function declines and these fibrotic pathways become activated, dysbiosis is an imbalance that favors pathobionts at the expense of symbionts by disrupting the intestinal ecosystem. It drives a surge in uremic toxins (UT), the ignition of systemic inflammation, the activation of immune pathways, and profound metabolic dysregulation. All of these converge to accelerate renal decline and amplify cardiovascular risk (Tan et al., 2022). The soaring prevalence, clinical complexity, and prohibitive cost of renal-replacement therapy, underscores an urgent need for innovative and pre-dialytic interventions (Sturov et al., 2023).

Encouragingly, the evidence demonstrates that targeted microbial supplementation not only improves renal function and optimizes lipid profiles but also has a beneficial effect on oxidative stress and inflammatory biomarkers in individuals suffering from CKD (Tan et al., 2022). Traditional Chinese medicine (TCM) and its purified extracts hold significant promise for the investigation and therapeutic application against a variety of diseases (Xiang et al., 2021; Guan et al., 2022). TCM has also demonstrated considerable efficacy in the treatment of CKD, capable of improving the metabolic pattern of intestinal flora and the intestinal barrier (Sun et al., 2022). TCM may be an important exogenous regulator of the crosstalk between the intestinal microbiota ecosystem and CKD, partly due to the mediating role of the targeted intervention of the microbiota (Sun et al., 2022). This review updates the therapeutic mechanisms, experimental evidence, and clinical progress of microecological preparations and TCM in improving CKD.

Before elaborating on these interventions, however, it is essential to establish the two fundamental metabolic pillars that underpin the gut–kidney axis and recur throughout this review: short-chain fatty acids (SCFAs) and uremic toxins (Li et al., 2026). SCFAs, primarily acetate, propionate, and butyrate, are fermentation end-products of dietary fibers by beneficial commensals. These metabolites serve pleiotropic protective functions: they maintain intestinal barrier integrity by regulating tight junction proteins, provide energy for colonocytes, modulate local and systemic immunity via G-protein-coupled receptor signaling, and exert anti-inflammatory effects that indirectly preserve kidney function. In stark contrast, uremic toxins, notably indoxyl sulfate and p-cresyl sulfate, are generated through microbial metabolism of tryptophan and tyrosine, respectively, by proteolytic pathobionts. Unlike SCFAs, these compounds are normally cleared by healthy kidneys, but as glomerular filtration rate declines in CKD, they accumulate systemically, activate NADPH oxidase and nuclear factor-κB pathways, induce oxidative stress and inflammatory cascades, and directly promote renal tubular injury and interstitial fibrosis. Thus, the bidirectional gut-kidney axis in CKD is mechanistically defined by a progressive shift: a decline in SCFA-producing symbionts and their protective metabolites, accompanied by an expansion of toxin-generating pathobionts and their deleterious byproducts. This metabolic imbalance not only drives CKD progression but also constitutes the primary therapeutic target for probiotics, prebiotics, synbiotics, fecal microbiota transplantation (FMT), and TCM, each of which, modulates this SCFA/uremic toxin equilibrium through distinct but complementary mechanisms. Before detailing these microbiota-based therapeutic strategies, however, several important methodological caveats must be acknowledged. First, the majority of mechanistic evidence discussed below originates from rodent models of CKD (including 5/6 nephrectomy, adenine-induced, and cisplatin-induced models), and causal relationships between specific microbial taxa and renal fibrosis remain largely inferential in humans. Second, throughout this review, we have explicitly separated preclinical from clinical evidence within each intervention section. Third, findings involving changes in microbiota composition or inflammatory markers are presented as mechanistic or correlative observations; they are not interpreted as direct evidence that an intervention slows CKD progression unless renal outcomes such as eGFR, serum creatinine, proteinuria, or hard clinical endpoints were directly measured. Fourth, the therapeutic responsiveness to these interventions may differ substantially across early versus advanced stages of CKD, and we have therefore indicated the CKD stage for each cited clinical study whenever the original report provided this information. Fifth, and critically, the therapeutic responsiveness to microbiota-targeted interventions is likely to differ substantially across the stage-specific responses, subtype-specific considerations, and caveats for animal-to-human translation.

## 2 Methods

As this review is narrative and integrative in nature, our literature search was guided by the goal of capturing the full spectrum of available evidence, rather than by the stringent criteria typically required for a meta-analysis. We mainly searched the following electronic databases: PubMed, Web of Science, and the Cochrane Library. The search period covered from database inception to May 31, 2026. The main search terms and their combinations were as follows: (“chronic kidney disease” OR “CKD” OR “end-stage renal disease” OR “end-stage kidney disease” OR “ESKD” OR “kidney failure”) AND (“gut microbiota” OR “gut microbiome” OR “intestinal flora” OR “dysbiosis”) AND (“probiotics” OR “prebiotics” OR “synbiotics” OR “fecal microbiota transplantation” OR “FMT” OR “traditional Chinese medicine” OR “TCM” OR “herbal medicine” OR “Chinese herbal formula”). We also manually screened the reference lists of retrieved articles and relevant reviews to identify additional studies. We included original research articles (both preclinical and clinical), systematic reviews, meta-analyses, and network meta-analyses published in peer-reviewed journals. For clinical studies, we prioritized randomized controlled trials (RCTs), but also included observational studies and case series. For preclinical studies, we included animal experiments that provided mechanistic insights into microbiota–kidney interactions. Two authors independently screened titles and abstracts, and discrepancies were resolved through discussion with a third author. The selection process was guided by relevance to the gut-kidney axis and microbiota-targeted interventions in CKD. Throughout the review, we distinguish between individual randomized controlled trials, network meta-analyses, observational studies, and case reports, and we explicitly note the limitations of indirect comparisons and network meta-analytic rankings where such evidence is discussed.

## 3 Characteristics of gut microbiota changes in CKD

The human gut microbiota is mainly composed of nonpathogenic microbial species. The gut microbiota composition varies among individuals and is readily influenced by genetics, the environment, age, and diet (Avuthu and Guda, 2022; Rinninella et al., 2019). Under physiological conditions, the gut microbiota exists in a balanced symbiosis with its host, orchestrating systemic homeostasis by regulating micronutrient biosynthesis, energy metabolism, maintaining intestinal barrier integrity, immunomodulation, and providing a strong defense against invading pathogens (Rinninella et al., 2019; Zhan et al., 2026).

A study of 1,436 CKD patients and 918 healthy controls shows that CKD is associated with an increase in Proteobacteria, Fusobacteria, *Escherichia-Shigella, Desulfovibrio*, and *Streptococcus*, and a decrease in *Roseburia, Faecalibacterium, Pyramidobacter*, and some *Prevotella* (Zhao et al., 2021b). Notably, emerging evidence indicates that the magnitude and direction of these microbial shifts vary across CKD subtypes: diabetic kidney disease exhibits a more pronounced expansion of *Escherichia-Shigella* and depletion of butyrate-producing genera compared with hypertensive nephropathy, whereas IgA nephropathy and membranous nephropathy display distinct enrichment patterns of Proteobacteria and Fusobacteria that correlate with disease activity and proteinuria levels, suggesting subtype-specific host-microbe interactions. These subtype-specific microbial signatures have important therapeutic implications: interventions that effectively modulate the gut microbiota in one CKD subtype may not yield comparable benefits in another, underscoring the need for subtype-stratified analyses in future clinical trials and caution against generalizing findings across the entire CKD spectrum. In ESKD, this pattern intensifies. Proteobacteria and *Streptococcus* proliferate further, accompanied by *Fusobacterium*, while *Prevotella, Coprococcus, Megamonas*, and *Faecalibacterium* decline (Zhao et al., 2021b). Metabolically, CKD patients show elevated trimethylamine-N-oxide and p-cresyl sulfate, along with reduced short-chain fatty acids. Due to heterogeneous methodologies, gut permeability could not be reliably quantified in this population. Wang et al. employed metagenomic sequencing technology and discovered that, compared with healthy individuals, 51% of the species in the intestinal microbiota of patients with ESKD were significantly altered (Wang et al., 2020). The genera *Eggerthella latae, Ruminococcus*, and *Fusobacterium* were enriched, while the genera *Prevotella, Clostridium*, and certain butyrate-producing bacteria were reduced.

The imbalance of intestinal flora not only affects the function of the intestinal barrier but also promotes systemic inflammatory responses and the accumulation of UT through the “gut-kidney axis” mechanism, thereby accelerating the progression of kidney damage (Narayan et al., 2004). Metagenomic analysis of 378 hemodialysis-dependent ESKD patients and 290 healthy controls revealed a disease-specific gut microbiota signature of 348 species (Zhang et al., 2023a). *Blautia, Dorea*, and Eggerthellaceae were found to be increased, while *Prevotella* and *Roseburia* showed a decrease. The functional profiles of these species encompassing antimicrobial resistance, aromatic compound catabolism, and bioactive metabolite synthesis are capable of predicting serum UT levels and tracking the progression of CKD in an independent non-dialysis cohort (Zhang et al., 2023a). This indicates that gut microbiota can influence the metabolism of CKD patients and predict the severity of their condition. In ESKD, the imbalanced gut microbiota creates a harmful metabolic environment, which increases morbidity and mortality. This makes the microbiome a key therapeutic target for reducing uremic toxicity (Wang et al., 2020). Recent comprehensive reviews have systematically consolidated these microbial signatures, reaffirming the consistent depletion of SCFA producers alongside enrichment of proteolytic pathobionts across diverse CKD cohorts, while also highlighting emerging evidence for functional metagenomic alterations and their correlation with clinical outcomes (Bloom et al., 2025; Jin et al., 2026; Sun et al., 2026; Zhao et al., 2025; He et al., 2025). The normal gut microbiota, along with their changes and impact on CKD, are shown in **Figure 1**.

**Figure 1:**
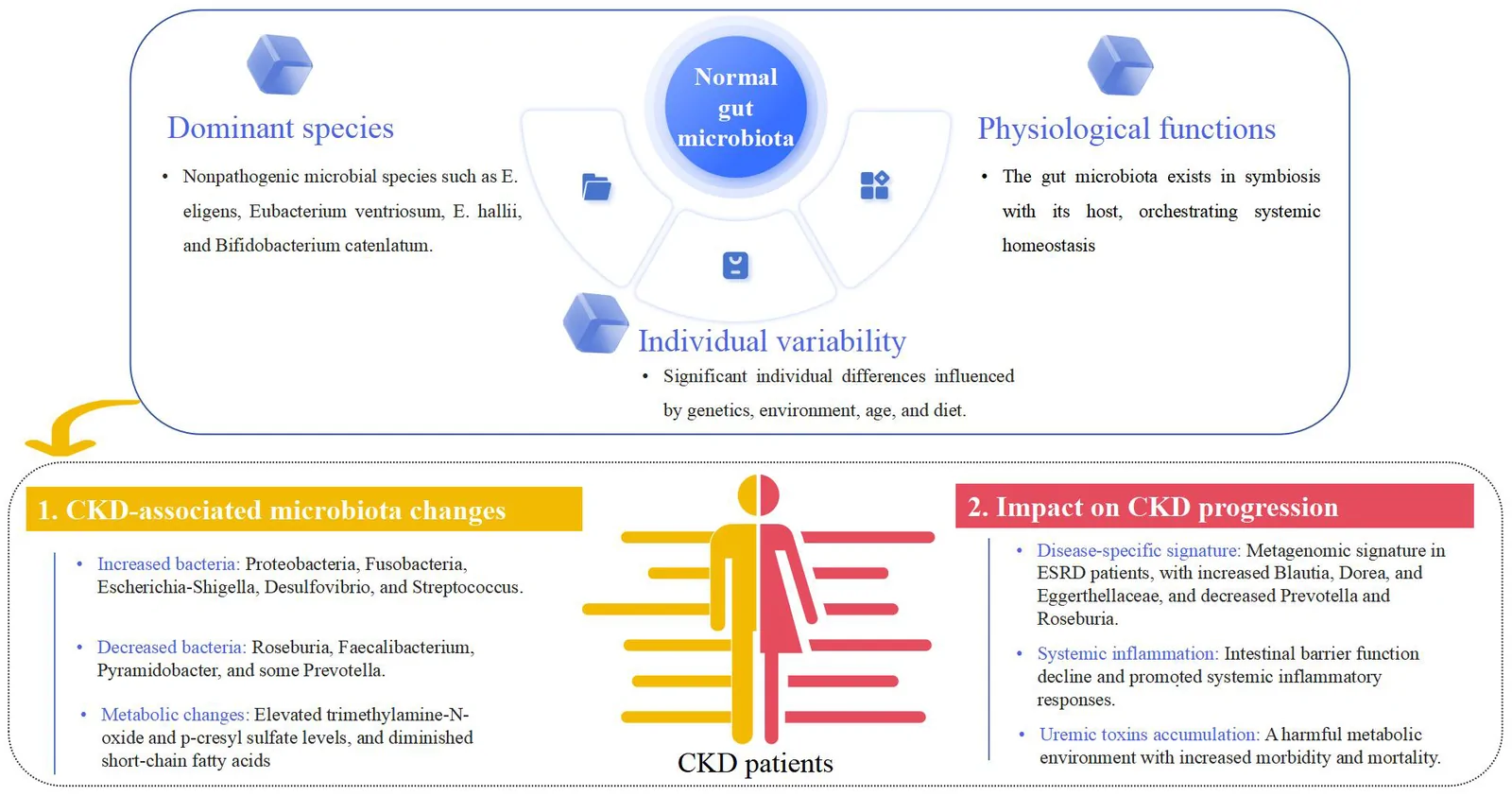
Normal gut microbiota, and their changes and impact on CKD. The gut microbiota in CKD exhibits significant individual variability shaped by genetics, environment, age, and diet, yet plays a fundamental symbiotic role in systemic homeostasis. Key disease-specific alterations include a rise in pathogenic taxa such as Proteobacteria and Escherichia-Shigella, alongside a depletion of beneficial short-chain fatty acid producers like Roseburia and Faecalibacterium, which drive uremic toxin accumulation, intestinal barrier dysfunction, and systemic inflammation that directly accelerates CKD progression and its associated morbidity.

## 4 Bidirectional regulation exists between CKD and gut microbiota

### 4.1 Role of CKD in gut microbiota

In CKD, persistently high urea levels promote the formation of UT, such as indoxyl sulfate, which triggers oxidative stress and inflammation, leading to extensive tissue damage and functional decline (Olivier et al., 2020). Metabolic disorders in CKD patients, such as the buildup of UT and medication use, can further alter intestinal pH, compromise the mucosal barrier, and disrupt immune homeostasis, creating a vicious cycle that exacerbates microbial imbalance (**Figure 2**). In addition, long-term dialysis treatment may also lead to the remodeling of the intestinal flora structure (Zhang et al., 2016). Wong et al. demonstrated that the CKD gut microbiota is enriched in taxa encoding indole and p-cresol biosynthetic enzymes and, concurrently, depleted of butyrate-producing species (Wong et al., 2014). As renal clearance declines in CKD, urea is redirected to the gut for excretion. In the intestinal lumen, it accumulates and is hydrolyzed by microbial urease into NH_3_, which rapidly converts to NH_4_OH, increasing the luminal pH (Lau et al., 2018). This alkaline shift selectively nurtures toxigenic pathogens while suppressing SCFA-producing commensals, contributing to systemic toxicity (Lau et al., 2018). SCFAs are beneficial for maintaining the intestinal barrier and the pH value of the intestinal lumen, as well as inhibiting the growth of pathogens (Cases et al., 2019). Fluid restriction, reduced physical activity, and low dietary fiber in CKD can lead to constipation, slow colonic transit, and prolonged fecal retention (Ramos et al., 2020). These conditions enhance the absorption of enteric toxins, exacerbate systemic toxin accumulation, and aggravate dysbiosis. Commonly prescribed nephrocentric pharmacotherapies including antibiotics, proton-pump inhibitors, laxatives, and statins significantly reshape both the taxonomic structure and functional profile of the gut microbiota (Weersma et al., 2020). An observational study revealed that 4 to 8 weeks of oral proton pump inhibitor therapy elicited a pronounced luminal expansion of *Lactobacillus* and *Streptococcus* species (Hojo et al., 2018).

**Figure 2:**
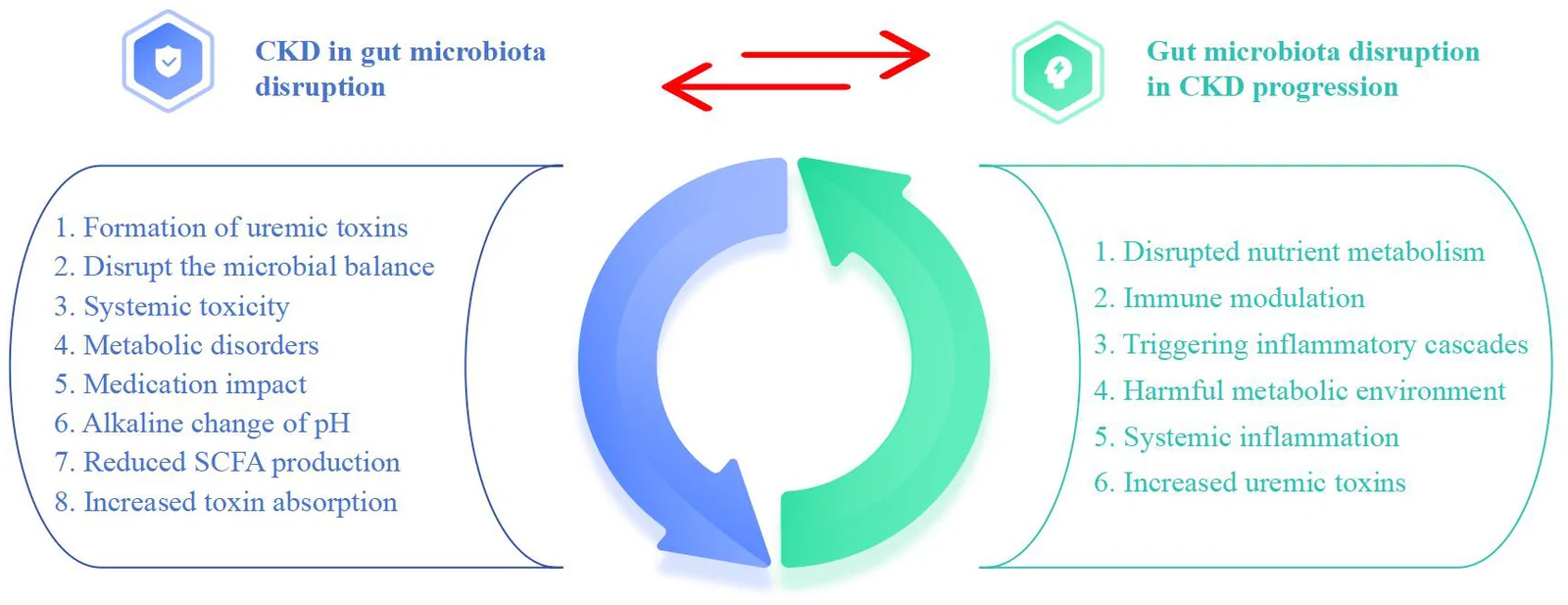
Bidirectional regulation between CKD and gut microbiota. Gut microbiota disruption in CKD drives progression through multiple interconnected pathways, including dysregulated nutrient metabolism, immune modulation, and the triggering of inflammatory cascades that create a harmful metabolic environment. This vicious cycle is visualized at the tissue level as progressive disruption of intestinal epithelial tight junctions (e.g., ZO-1 and occludin), increased paracellular permeability, and translocation of luminal endotoxins and uremic toxins into the portal circulation. Concurrently, in the renal parenchyma, these insults are depicted as tubular epithelial cell atrophy, peritubular capillary rarefaction, and interstitial infiltration by inflammatory cells, which together establish a self-perpetuating loop. This vicious cycle leads to systemic inflammation, elevated uremic toxin levels, impaired short-chain fatty acid production, and increased intestinal toxin absorption, further exacerbating microbial imbalance and CKD progression.

Collectively, a confluence of endogenous perturbations and exogenous exposures converges to reshape the gut microbiota in patients with CKD. Based on the gut–kidney axis, the interaction between kidney disease and the gut microbiota involves four key disruptions during CKD onset and progression: compositional dysbiosis, increased gut-derived UT, a compromised intestinal barrier, and systemic endotoxemia (Chen et al., 2019).

### 4.2 Role of gut microbiota in CKD

The gut microbiota orchestrates nutrient metabolism, strengthens the host’s defense against infection, and modulates immune responses. It establishes bidirectional communication with distant organs, significantly influencing the development of obesity, cardiovascular disease, diabetes, and kidney disorders (Nesci et al., 2023; Liu et al., 2021). Dysbiosis of intestinal microbiota caused by various reasons can lead to the compensatory entry of urea into the intestinal lumen, exacerbate intestinal barrier injury, trigger the body’s inflammatory response, promote the production of toxic substances, and accelerate renal functional and structural damage as well as the progression of CKD. (**Figure 3**) (Zhu et al., 2023).

**Figure 3:**
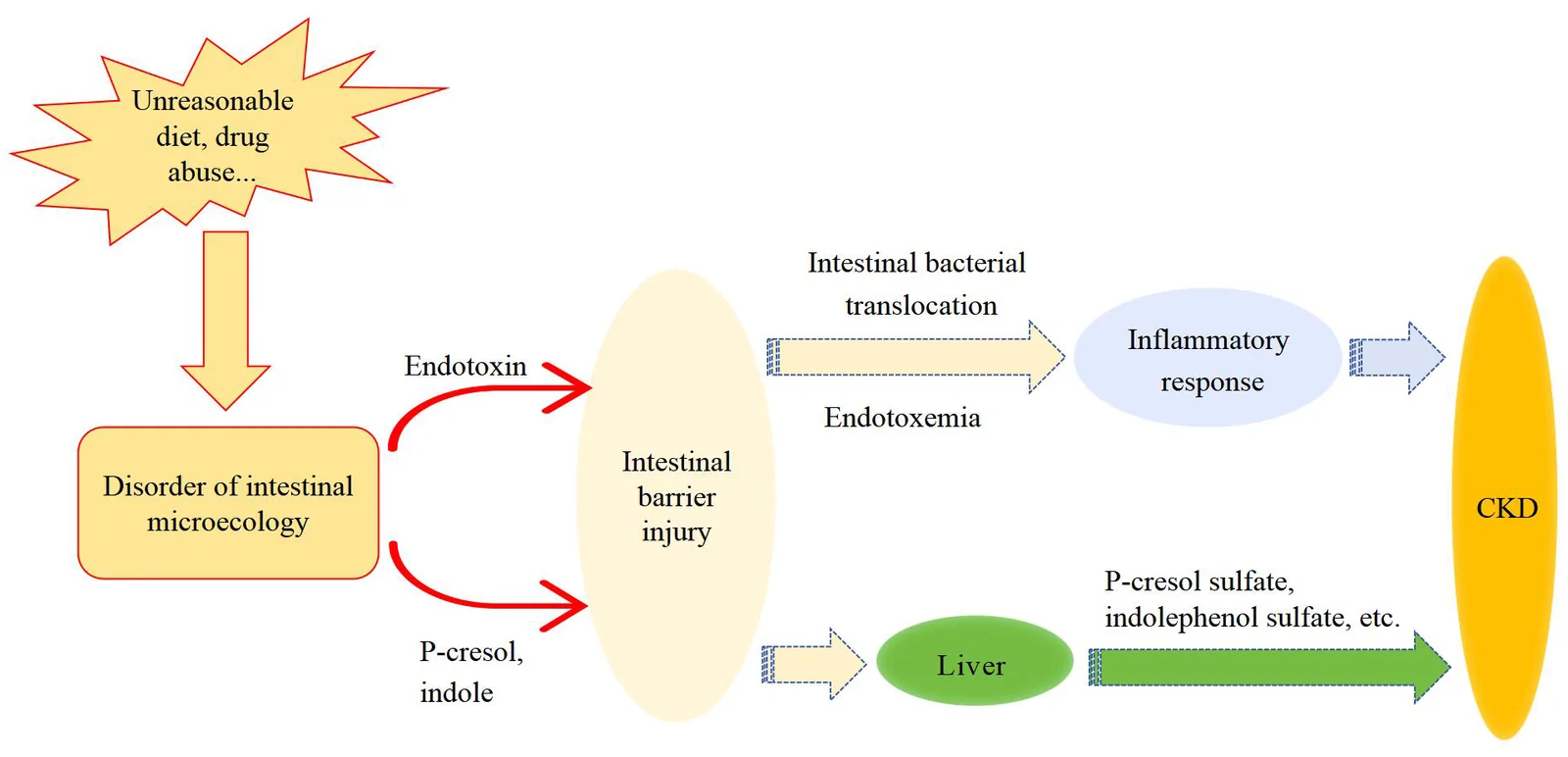
Role of intestinal microecology in CKD. Unreasonable diet and drug abuse disrupt intestinal microecology, leading to elevated levels of harmful metabolites such as endotoxin, p-cresol, and indole, which trigger intestinal barrier injury, inflammatory responses, and bacterial translocation. At the microscopic level, this is manifested as loss of brush border microvilli, fragmentation of intercellular adherens junctions, and overt epithelial denudation, allowing luminal bacteria and their byproducts to cross the mucosa. In the kidney, these translocated toxins are depicted as inducing oxidative stress–mediated vacuolization of proximal tubular cells, activation of resident fibroblasts, and early deposition of extracellular matrix proteins, all of which collectively drive progressive nephron loss. These pathological events culminate in endotoxemia and accumulation of uremic toxins like p-cresol sulfate and indoxyl sulfate, thereby accelerating the progression of CKD.

Pathobionts and their toxins, breaching a compromised mucosal barrier, ignite inflammatory cascades that blunt the efficacy of renal-replacement therapy (Ritz, 2011). In 2011, Meijers and Evenepoel first articulated the gut-kidney axis, positing a bidirectional nexus in which renal excretory competence and intestinal microbial metabolism operate as an integrated physiological unit (Meijers and Evenepoel, 2011). CKD alters the metabolic environment, which in turn reshapes the microbiome. In response, the damaged microbiota increases the production of harmful metabolites, including endotoxins (Sumida et al., 2023). These endotoxins, when entering the bloodstream, exacerbate systemic inflammation and accelerate disease progression (Sumida et al., 2023). Building on this paradigm, Pahl et al. subsequently introduced the “CKD-colon axis”, underscoring the inseparable reciprocity between CKD pathogenesis and the gut microbiota (Pahl and Vaziri, 2015). CKD alters microbial composition and metabolic activity. Consequently, the dysbiotic community increases the production of UT, which causes additional renal damage, perpetuating a harmful pathophysiological cycle. The bidirectional nature of this crosstalk, wherein renal dysfunction reshapes the intestinal microbial ecosystem and the altered microbiome in turn accelerates renal injury has been comprehensively delineated in several authoritative reviews that integrate metagenomic, metabolomic, and clinical translational evidence (Bloom et al., 2025; Jin et al., 2026; Sun et al., 2026; Zhao et al., 2025; He et al., 2025).

## 5 Microecological preparations for CKD

### 5.1 Definition and classification of microecological preparations

Microecological preparations (a term used in the Chinese and broader Asian literature to encompass probiotics, prebiotics, synbiotics, and related microbiome-modulating products) consist of live or inactivated microorganisms and/or their metabolites. They can re-establish intestinal homeostasis by promoting commensal flora and suppressing pathogens, ultimately enhancing host health and performance. Taxonomically, they are classified as probiotics, prebiotics, or synbiotics based on composition and mechanism (Wang et al., 2023b). For clarity, throughout this review we use the more widely recognized terms, probiotics, prebiotics, and synbiotics, when referring to specific intervention categories, while using “microecological preparations” as an umbrella term where aggregation across categories is intended.

In 2014, the Food and Agriculture Organization of the United Nations and the WHO defined a probiotic as “live microorganisms which, when administered in adequate amounts, confer a health benefit on the host” (Hill et al., 2014). The current consensus is that probiotics are viable bacteria or yeasts that colonize the gut and restructure the intestinal microbiome to suppress pathogens, reinforce barrier integrity, and modulate mucosal immunity. Thereby, they serve as evidence-based adjuncts for a spectrum of gastrointestinal disorders while promoting systemic well-being (Kim et al., 2019; Wieërs et al., 2019). Beneficial probiotic taxa consistently linked to host health include the lactic-acid-producing genera *Lactobacillus* (e.g., *L. rhamnosus, L. casei*), *Lacticaseibacillus, Limosilactobacillus, Ligilactobacillus, and Bifidobacterium* (e.g., *B. lactis, B. longum, B. breve, B. bifidum*). The enteric strain *Escherichia coli* Nissle 1917 is also well-documented (Parhizkar et al., 2025). These organisms predominantly colonize the gastrointestinal tract, especially the ileum and colon. There, they ferment dietary fibers, generate short-chain fatty acids, reinforce barrier integrity, and modulate systemic immunity (Parhizkar et al., 2025).

Prebiotics were first defined in 1995 as substances used to manipulate microorganisms within a host to improve measurable health outcomes. The definition of prebiotics released in 2017 was updated to “a substrate that is selectively utilized by host microorganisms, conferring a health benefit” (Gibson et al., 2017). Prebiotics are no longer merely regarded as boosters for the growth of Bifidobacteria and lactic acid bacteria. Instead, they are now considered to have an impact on the metabolic and physiological readings of the entire system, including the intestinal tract, urogenital tract, oral-nasal area, and the microbial community on the skin (Sanders et al., 2019). Prebiotics encompass non-digestible, fermentable carbohydrates, particularly fructo-oligosaccharides, galacto-oligosaccharides, and dietary fibers such as inulin and resistant starch. These selectively stimulate the proliferation and metabolic activity of beneficial gut microbes (e.g., Bifidobacterium and Lactobacillus spp.) and thereby foster a microbiome profile associated with improved skin and systemic health (Parhizkar et al., 2025).

The initial definition of synbiotics is “probiotics and prebiotics that beneficially affect the host”. In 2020, the International Scientific Association for Probiotics and Prebiotics (ISAPP) refined its definition as “a mixture comprising live microorganisms and substrate(s) selectively utilized by host microorganisms that confers a health benefit on the host” (Kleerebezem and Führen, 2024). Synbiotic intake broadly improves anthropometric, cardiometabolic, and inflammatory indices, eradicates *Helicobacter pylori*, and alleviates symptoms in conditions such as atopic dermatitis and colorectal cancer. However, the rational design of most synbiotic formulations and their superiority over individual probiotics or prebiotics remain insufficiently substantiated. The synbiotic formulations examined included Lactocare^®^ (containing *L. casei, L. acidophilus, L. rhamnosus, L. bulgaricus, B. breve, B. longum, S. thermophilus* plus fructo-oligosaccharides), a six-strain blend with fructo-oligosaccharides (*S. thermophilus, E. faecium, L. plantarum, L. acidophilus, B. lactis, B. longum* plus fructo-oligosaccharide), *B. breve* Yakult with galacto-oligosaccharide, *B. longum* with Synergy 1, *L. plantarum* SC-5 with tyrosol, *L. plantarum* YW11 with lactulose, and *B. animalis* lactis with fructo-oligosaccharides (Saedi et al., 2024). A meta-analysis found that the use of probiotics alone could not improve the clinical indicators of metabolic dysfunction-associated steatotic liver disease. In contrast, synthetic preparations significantly reduced liver steatosis and tumor necrosis factor-α (TNF-α) levels, increased HDL-C levels, and improved the inflammatory markers (ALT and AST) of hepatocytes. About 12 weeks is the ideal intervention cycle for patients with metabolic dysfunction-associated steatotic liver disease (Wu et al., 2024a).

### 5.2 Microecological preparations in treating CKD

Dysbiosis of the intestinal flora leads to an increase in toxic metabolic products. Meanwhile, the decline in renal function slows down the clearance of toxins. Eventually, this results in the retention of toxic metabolic products from the intestinal flora in the body, which becomes an important source of UT (Ramezani et al., 2016). In human ESKD, microbiota drives renal injury by overproducing UT and secondary bile acids; Eggerthella lenta and Fusobacterium nucleatum are key culprits, whereas Bifidobacterium animalis counters this effect (Wang et al., 2020). UT are excessively produced during CKD as products of food metabolism or as a result of the loss of renal function, which have a negative impact on the kidneys and other organs (Plata et al., 2019). Microecological preparations can effectively reduce UT levels, increase the production of SCFAs, and restore normal mucosal barrier function, thereby reducing inflammatory responses (Mahmoodpoor et al., 2017). CKD-associated gut dysbiosis drives the accumulation of UT, accelerating disease progression (Amini Khiabani et al., 2023). The improvement effect of microecological preparations on the structure and function of the kidneys has been found in multiple preclinical and clinical studies, with renal functional outcomes (serum creatinine, eGFR, proteinuria) directly measured in clinical trials as noted below. Emerging microecological preparations for CKD are shown in **Figure 4**.

**Figure 4:**
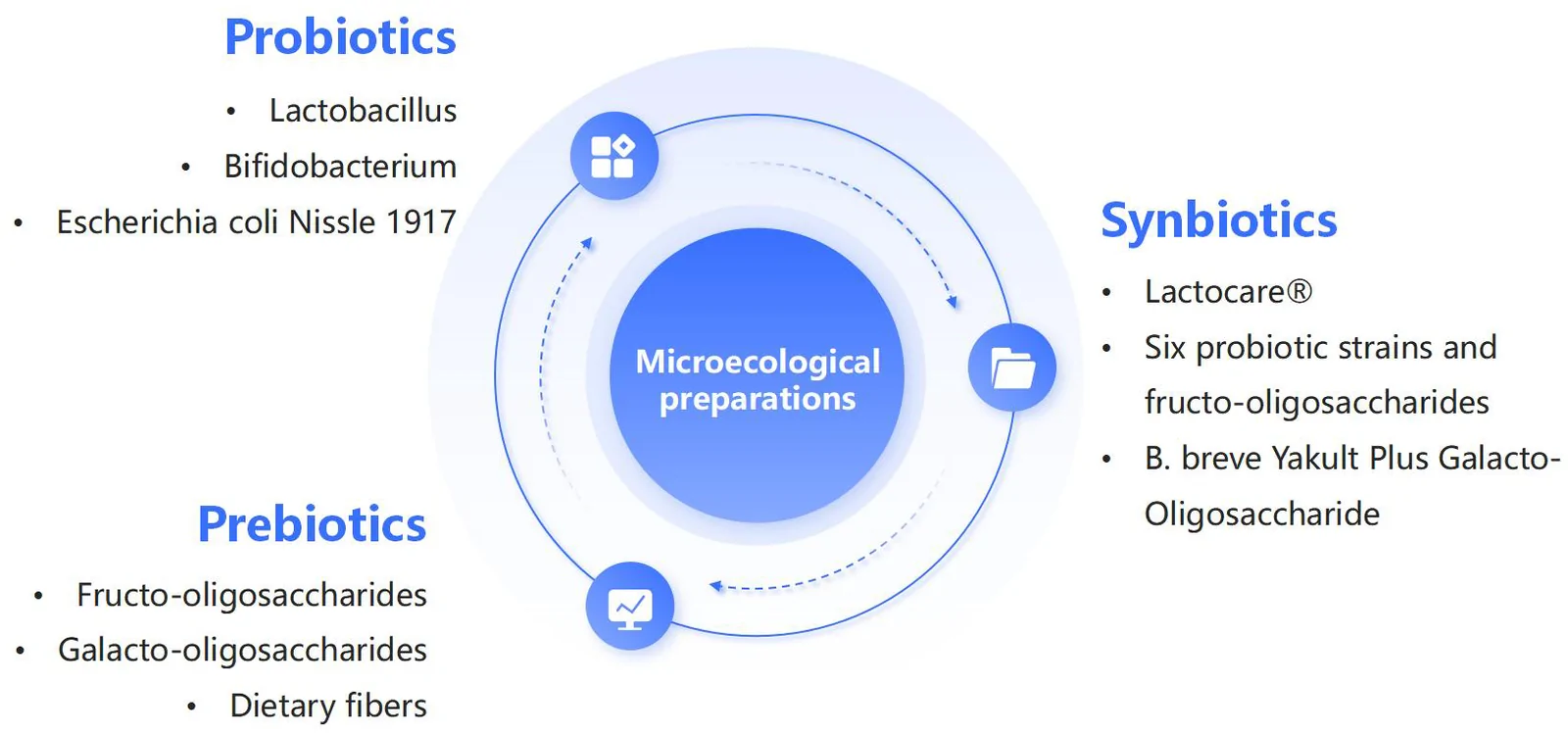
Emerging microecological preparations for CKD. Probiotics, along with prebiotics, have been widely investigated for their beneficial effects on gut microbiota. Synbiotic formulations represent synergistic approaches that simultaneously provide live beneficial bacteria and their growth substrates.

#### 5.2.1 Preclinical evidence

In adenine-induced CKD rats, dysbiosis manifests as reduced diversity and overgrowth of indole-producing *Clostridium*, whereas synbiotics restore balance (Yang et al., 2021). The translational relevance of these findings to human CKD subtypes, however, remains incompletely defined; for instance, DKD patients exhibit more prominent endotoxemia-driven inflammatory responses to synbiotic intervention compared with non-diabetic CKD, whereas IgAN patients show greater variation in therapeutic response that may be modified by baseline gut permeability and circulating IgA levels. The translational relevance of these findings to human CKD subtypes remains incompletely defined and requires explicit qualification. For instance, diabetic kidney disease patients exhibit more prominent endotoxemia-driven inflammatory responses to synbiotic intervention compared with non-diabetic CKD, likely due to the compounding metabolic burden of hyperglycemia and insulin resistance. IgAN patients show greater variation in therapeutic response that may be modified by baseline gut permeability, circulating IgA levels, and the degree of gut-associated lymphoid tissue involvement. Focal segmental glomerulosclerosis (FSGS) patients, by contrast, may derive benefit primarily through FMT-mediated metabolic correction and dyslipidemia improvement rather than direct immunomodulation. These subtype-specific differences preclude straightforward generalization of findings from one CKD subtype to another, and future trials should be designed with subtype-stratified analyses to identify differential responders. In 5/6-nephrectomized rats, oral administration of *Lactiplantibacillus paraplantarum* KCCM 11826P, which was equipped with a polyP gene cluster that enhanced intracellular phosphate sequestration and suppressed indole-producing bacteria, hints at the strain as a dual-action probiotic phosphate binder and UT reducer in CKD (Moon et al., 2022). In 5/6-nephrectomized mice, 14-week daily administration of the local strain *Lactobacillus rhamnosus* L34 not only significantly reduced circulating trimethylamine-N-oxide and indoxyl sulfate but also alleviated inflammation (with lower serum endotoxin [P = 0.03] and TNF-α [P = 0.01] versus untreated controls) and mitigated kidney fibrosis (P < 0.01), serum creatinine, proteinuria, and structural renal damage (Tungsanga et al., 2022). In adenine-CKD mice, 4 weeks of treatment with *Lactobacillus acidophilus* KBL409 reduced renal fibrosis, restored a disrupted intestinal barrier, and improved kidney function, as shown by lower serum creatinine and albuminuria (Kim et al., 2022).

Oral administration of *Lactobacillus rhamnosus* GKLC1 reversed cisplatin-induced CKD in mice, resulting in dose-dependent reductions in the kidney weight index, histopathological damage, serum BUN, and creatinine. *In vitro*, it blocked cisplatin-induced nephrotoxicity by suppressing MAPK/NF-κB/COX-2 inflammation, p53/Bax/Caspase-3 apoptosis, and STAT3-mediated fibrosis (Tsai et al., 2022). *Lactobacillus johnsonii* BS15 protected against fluoride-induced renal injury by restoring colonic microbiota balance, suppressing four dysbiosis-linked keystone OTUs, and reversing oxidative stress and tubular damage (Xin et al., 2021). Critically, an independent large-scale study involving 480 participants across CKD stages 1-5 identified a taxonomic chain Bacilli-Lactobacillales-Lactobacillaceae-*Lactobacillus*-*Lactobacillus johnsonii* that strongly correlated with CKD progression, with *Lactobacillus johnsonii* abundance declining progressively in parallel with kidney function deterioration (Miao et al., 2024). Serum indole-3-aldehyde (IAld), a microbial tryptophan metabolite whose levels strongly negatively correlate with serum creatinine, was found to be decreased in both CKD rats and late-stage CKD patients; treatment with IAld dampened kidney injury by suppressing AhR signaling, and *Lactobacillus johnsonii* supplementation attenuated renal lesions through increasing serum IAld and inhibiting AhR activation, a mechanism that was partially abolished in AhR-deficient mice and cultured HK-2 cells. Beyond the Lactobacillus genus, the probiotic strain *Lactobacillus casei* Zhang exerted renoprotective effects in both acute kidney injury and CKD models, attenuating renal tubular damage, reducing interstitial fibrosis, and preserving renal function through modulation of gut microbiota composition and suppression of systemic inflammatory responses, highlighting the broad therapeutic potential of Lactobacillus-based probiotics across the spectrum of kidney diseases (Zhu et al., 2021a). These findings establish the *Lactobacillus johnsonii*-IAld-AhR axis as a promising microbiota-dependent therapeutic target for CKD reversal, with potential translational implications for probiotic strain selection. While these mechanistic insights are predominantly derived from rodent models of CKD, correlative human studies have largely confirmed the directionality of these effects on uremic toxin reduction and inflammatory markers, although rigorous causal proof in humans, such as through targeted microbial depletion or gnotobiotic approaches, remains methodologically challenging and ethically constrained.

A comprehensive mechanistic framework has recently delineated that CKD-associated microbial dysbiosis impairs intestinal epithelial barrier integrity, depleting beneficial bacteria such as *Lactobacillus reuteri* and *Bifidobacterium animalis* while expanding pathogens including *Eggerthella lenta* and *Fusobacterium nucleatum*; this compositional shift reduces SCFA production and elevates uremic toxins such as indoxyl sulfate, indole-3-acetic acid (IAA), and trimethylamine-N-oxide, thereby exacerbating renal inflammation and fibrosis (Liu et al., 2026b). Conversely, probiotic strains including *Parabacteroides goldsteinii, Lactobacillus johnsonii, Bacteroides ovatus*, and *Bacteroides fragilis* have demonstrated attenuation of renal fibrosis, mediated by microbial-derived metabolites such as IAA, TMAO, indole-3-aldehyde, hyodeoxycholic acid, and 1,5-anhydroglucitol. *Akkermansia muciniphila*’s mucin-foraging activity reinforces the gut barrier to curtail toxin entry and systemic exposure, with its higher abundance correlating with diminished inflammation and metabolic/inflammatory disease risk, while its depletion is conversely linked to intestinal permeability and exacerbated inflammation (Pei et al., 2023). UT p-Cresyl sulfate and indoxyl sulfate are taken up into renal tubular cells via organic anion transporters. Once inside, both toxins activate NADPH oxidase, generating excess reactive oxygen species (ROS). Through organic anion transporters, p-cresyl sulfate enters renal tubular cells and activates Nox4/p22^phox, leading to excess ROS generation, which in turn induces a redox burst and upregulates inflammatory cytokines along with active TGF-β1, ultimately provoking oxidative damage, fibrosis, and tubular cell death, all of which are preventable by Nox4 knockdown or transport inhibition (Watanabe et al., 2013).

#### 5.2.2 Clinical evidence

In ESKD patients, indole producers are normal, yet indoxyl sulfate accumulates because of failed renal clearance. Meanwhile, SCFA-producers *Faecalibacterium* and *Roseburia* decline, and *Fusobacterium, Shewanella*, and *Erwinia* emerge (Yang et al., 2021). Anegkamol et al. demonstrated that resistant maltodextrin outperforms inulin and chitosan oligosaccharide by concurrently inhibiting inflammation, strengthening the intestinal barrier, and selectively enriching the keystone genera *Lactobacillus, Bifidobacterium, Akkermansia*, and *Roseburia* (Anegkamol et al., 2023). Probiotic intervention in patients with mild renal insufficiency (stage 3a of CKD) not only selectively restored fecal Lactobacillales and Bifidobacteria but also consequently reduced urinary indican and 3-methylindole to normal levels, along with a simultaneous decrease in CRP (Simeoni et al., 2019). After 12 weeks of synbiotics (*Lactobacillus acidophilus, Lactobacillus casei*, and *Bifidobacterium lactis*, 32 billion CFU/day plus 3.2 g inulin), CKD patients showed a significant 12% increase in eGFR alongside a 21.5% reduction in serum indoxyl sulfate (P < 0.001) and a 39.5% decrease in high-sensitivity CRP (P < 0.001), while p-cresyl sulfate and trimethylamine N-oxide remained unchanged, collectively indicating improved kidney function and reduced inflammation, providing direct evidence of improved kidney function in the clinical setting (Mitrović et al., 2023). In a probiotics-supplemented low-protein diet for CKD, adding *Bifidobacterium longum* plus *Lactobacillus reuteri* to a low-protein diet halted the rise of indoxyl sulfate and other pro-atherogenic toxins observed in placebo-treated advanced CKD patients. Meanwhile, probiotic supplementation led to a downward trend in these microbial metabolites (De Mauri et al., 2022). Probiotics restructured the gut microbiota in non-diabetic HD patients by enhancing Bacteroidaceae and Enterococcaceae while suppressing several UT-linked taxa. While these findings are encouraging, the safety of probiotics in hemodialysis patients with central venous catheters or other immunocompromised states requires dedicated attention, as cases of probiotic-associated bloodstream infections have been reported in vulnerable populations. This led to measurable reductions of indole-3-acetic acid-O-glucuronide and other retention solutes without altering overall diversity (Liu et al., 2020). In hemodialysis patients, three-month intake of the *Bifidobacterium bifidum* BGN4 + *B. longum* BORI blend (2 × 10^10^ CFU b.i.d.) shifted the gut microbiota toward higher SCFAs production. This shift was paralleled by a marked fall in serum calprotectin and LPS-stimulated interleukin-6 (IL-6), an expansion of CD4^+^CD25^+^ Tregs, and a contraction of pro-inflammatory CD14^+^CD16^+^ monocytes (Choi et al., 2022).

In hemodialysis patients, four weeks of resistant starch type-2 (RS2)-enriched cookies (16 g/day) reworked the gut microbiota, upregulated SCFAs producers including *Roseburia* and *Ruminococcus gauvreauii*, and downregulated the pro-inflammatory *Dialister* (Kemp et al., 2021). In hemodialysis patients, resistant starch-enriched cookies significantly reduced plasma indoxyl sulfate (IS) (Esgalhado et al., 2020), an effect accompanied by significant up-regulation of Nrf2 mRNA and its downstream antioxidant NQO1, without affecting NF-κB expression (Esgalhado et al., 2020). Three months of 10 g/day inulin-type fructans supplementation in continuous ambulatory peritoneal dialysis patients shifted the gut microbiota by increasing the Firmicutes/Bacteroidetes ratio (p = 0.049) (Xiong et al., 2023). Inulin-type fructans significantly reduce fecal indole (-10.1 μg/g versus +13.3 μg/g with placebo; P = 0.040) and lower the levels of the indole-producer *Bacteroides thetaiotaomicron.* However, they leave fecal p-cresol, serum indoxyl sulfate and p-Cresyl sulfate, as well as their urinary or dialytic removal, unchanged (Li et al., 2020b). Elevated serum uric acid is an independent risk factor for increased mortality in ESKD patients. Inulin-type prebiotics (a type of fermentable dietary fiber) reduce serum uric acid by approximately 10% without increasing renal clearance. Instead, they augment fecal UA degradation and enrich purine-catabolizing *Clostridium* and related *Firmicutes*, thereby linking gut microbial purine metabolism to systemic UA control (He et al., 2022). β-glucan markedly lowered free indoxyl sulfate at 8 weeks (p = 0.003) and 14 weeks (p < 0.001), free p-cresyl sulfate at 14 weeks (p = 0.006), and both total and free p-cresyl glucuronide and indoxyl-3-acetic acid at 14 weeks (all p < 0.001) (Ebrahim et al., 2022). Synbiotic therapy in predialysis adult participants with CKD (eGFR = 10–30 ml/min per 1.73 m^²^) decreased serum p-cresyl sulfate and favorably modified the stool microbiome, particularly with enrichment of *Bifidobacterium* and depletion of *Ruminococcaceae*. However, it did not significantly reduce serum indoxyl sulfate (Rossi et al., 2016). This stage-specific observation, predialysis patients showing partial response, contrasts with the more consistent eGFR improvements reported in early-stage CKD, suggesting that the therapeutic window for microbiota modulation may narrow as renal function declines, likely due to the compounding effects of uremic metabolite accumulation, dietary restrictions, and polypharmacy that render the gut ecosystem progressively refractory to intervention. Synbiotic and probiotic supplementation both significantly reduced serum endotoxin (p = 0.036) compared to the placebo in hemodialysis patients (Haghighat et al., 2020). Synbiotics resulted in the most significant reduction of high-sensitivity CRP and IL-6 (both p < 0.001 vs placebo). Meanwhile, both synbiotics and probiotics reduced anti-HSP70 antibodies (p = 0.002 and p = 0.013, respectively) in hemodialysis patients (Haghighat et al., 2020). 60 days of synbiotics (Lactobacillus acidophilus and Bifidobacterium longum 5x10^9^ CFU) significantly improved constipation symptoms (p = 0.006) and constipation-related quality of life (p = 0.001) in chronic hemodialysis patients with gastrointestinal complaints, difficulty defecating, feces of hard consistency, or a bowel movement frequency of fewer than three times per week. This indicates enhanced gut motility and mucosal comfort without measured changes in toxin load (Lydia et al., 2022).

Recent network-meta-analytic evidence indicates that prebiotic, probiotic, or synbiotic interventions in stage 3–5 CKD patients beneficially remodel the gut microbiota and lower free/total p-cresyl sulfate and indoxyl sulfate. Although additional trials are required to elucidate the mechanisms of microbiota modulation and its downstream effects on the enteric bacterial community (Cedillo-Flores et al., 2025). A network meta-analysis in ESKD patients undergoing dialysis revealed that prebiotics were superior to probiotics and synbiotics in lowering indoxyl sulfate, BUN, IL-6, and TNF-α, whereas synbiotics were more effective than the other interventions in reducing both endotoxin and CRP (Yu et al., 2022). These findings, however, are derived from indirect comparisons and should be interpreted with caution, as the network meta-analysis included a limited number of trials per comparison and exhibited substantial heterogeneity in intervention protocols and patient characteristics. Another network meta-analysis of randomized clinical trials showed that prebiotics reduced hs-CRP (MD -2.06) and TNF-α (MD -2.65). Synbiotics also lowered hs-CRP (MD -2.01) in CKD patients, whereas a separate network meta-analysis of RCTs reported that probiotics exerted a beneficial effect on serum creatinine reduction (Tan et al., 2022). Notably, the same network meta-analysis by Tan and colleagues found no significant effect of any intervention on uremic toxins, underscoring the inconsistency across different analytical approaches and the need for cautious interpretation of treatment rankings. These discordant findings highlight that network meta-analytic evidence should be considered hypothesis-generating rather than definitive. In patients with chronic coronary syndrome, higher IL-6 levels independently predict cardiovascular events across all CKD stages, and the risk rises as kidney function declines (Batra et al., 2021). Therefore, the inflammatory state has an impact on the long-term prognosis of CKD patients. A comprehensive synthesis of preclinical and clinical evidence has reaffirmed that probiotic and synbiotic interventions consistently reduce uremic toxins, apoptosis, inflammation, and oxidative stress while restoring intestinal integrity and modulating microbial diversity; these positive effects have been observed across different CKD stages, with improvements in creatinine levels, uremic parameters, and abdominal discomfort, alongside reduced serum endotoxin and favorable modulation of gut microbial composition (Ribeiro et al., 2025). Peritoneal dialysis (PD), while offering advantages over in-center hemodialysis including home-based care and preservation of residual kidney function, remains an understudied context for probiotic interventions; although the gut microbiota is increasingly recognized as a determinant of PD outcomes including systemic inflammation, peritonitis risk, and metabolic disturbances, specific clinical data on probiotic efficacy in PD patients are limited and warrant dedicated investigation (Stepanova, 2024). To facilitate rapid extraction of key clinical evidence, we have summarized the major clinical studies on probiotics, prebiotics, synbiotics, FMT, and TCM in CKD in **Supplementary Table 1**, which includes study design, CKD stage or dialysis status, intervention and dose, treatment duration, main renal and metabolic outcomes, adverse events, and major limitations. FMT case reports and exploratory trials are clearly distinguished from randomized controlled trials to enable appropriate interpretation of evidence levels. When interpreting the clinical evidence for microecological preparations, it is essential to stratify findings by CKD stage and dialysis status, as therapeutic responses differ substantially across these populations. These stage-dependent and dialysis-dependent differences should be considered when evaluating the overall evidence base and designing future trials. The mechanisms of microecological preparations in treating CKD are shown in **Figure 5**, which conceptually illustrates the transition from healthy intestinal villi with intact tight junctions and cuboidal renal tubular epithelia to the dysbiotic state featuring eroded mucosa, endotoxin leakage, and tubular atrophy with interstitial edema, and subsequently to the restored state after intervention, depicted by repaired barrier structures, reduced oxidative stress, and preserved nephron architecture.

**Figure 5:**
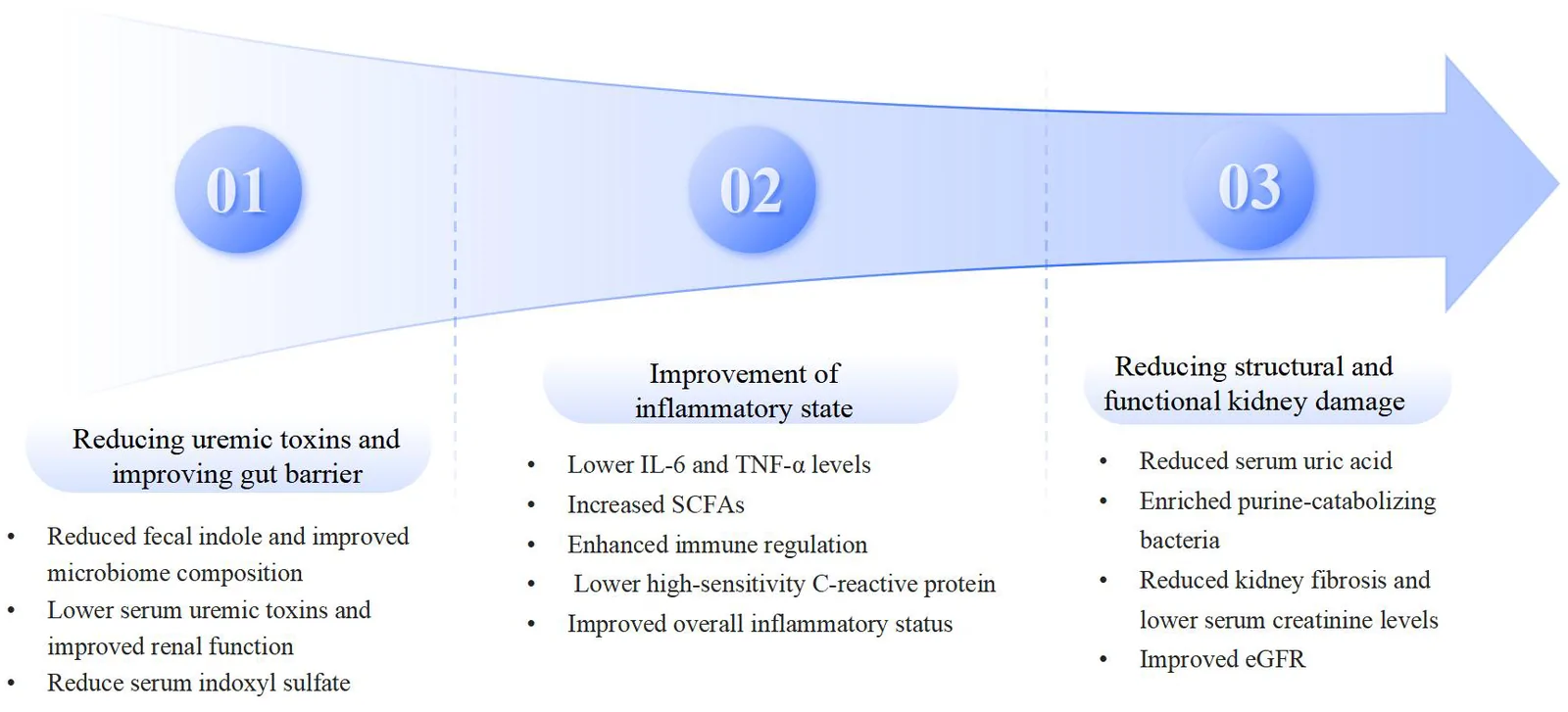
Mechanisms of microecological preparations in treating CKD. Microecological preparations mitigate chronic kidney disease progression through a triad of mechanisms: they reduce uremic toxins by reshaping gut microbiota and lowering indoxyl sulfate, alleviate systemic inflammation via short-chain fatty acid-mediated immune modulation, and attenuate kidney fibrosis while improving renal function and estimated glomerular filtration rate. These effects are best understood through pathological icons: in the gut, probiotics/prebiotics restore the mucin layer, reinforce tight junction strands (visualized as continuous “zipper-like” structures between adjacent enterocytes), and reduce paracellular flux of bacterial endotoxins. In the kidney, these interventions translate into reduced tubular cell apoptosis (depicted as decreased caspase-3-positive nuclei), diminished interstitial collagen deposition (shown as thinner blue-stained fibrotic septa), and preserved glomerular capillary loops. These coordinated effects, achieved by enriching purine-catabolizing bacteria and reducing key pro-inflammatory cytokines, position gut-targeted interventions as a promising strategy to delay CKD progression.

## 6 FMT and CKD

FMT is a valuable tool for uncovering the role of gut microbiota in the pathological processes (Belvoncikova et al., 2022). FMT restores gut eubiosis along the dysregulated gut-kidney axis, thereby reducing UT load and systemic inflammation and presenting a promising microbiota-based therapy to slow CKD progression (**Figure 6**) (Bian et al., 2022). FMT restores microbial diversity, reshapes the gut community toward a healthy profile, curbs p-cresyl sulfate accumulation, and improves glucose tolerance without altering renal function (Barba et al., 2020). FMT re-establishes a balanced gut microbiota in CKD (Caggiano et al., 2023). Thereby, it lowers dysbiosis-driven UT, dampens systemic inflammation, and mitigates associated cardiovascular and renal damage (Caggiano et al., 2023). However, these putative benefits remain largely extrapolated from preclinical models and mechanistic reasoning, with limited clinical validation to date. In human CKD subtypes, FMT appears to exert differential effects: in IgAN, the therapeutic benefit is primarily mediated through modulation of the gut-associated lymphoid tissue and reduction of galactose-deficient IgA1, whereas in FSGS, FMT-mediated metabolic correction and dyslipidemia improvement appear more central to its renoprotective action; in DKD, the anti-inflammatory and insulin-sensitizing effects of FMT are more prominent, likely due to the concurrent metabolic burden of hyperglycemia and insulin resistance. Furthermore, the stage of CKD at which FMT is administered may critically influence outcomes: the available human evidence, though preliminary, suggests that FMT may be more effective in stage 3–4 CKD than in stage 5 or dialysis-dependent patients, possibly because the profound uremic milieu and extensive microbial remodeling in advanced disease render the gut ecosystem less receptive to engraftment and sustained microbial reconstitution. This stage-dependent heterogeneity should be explicitly considered when interpreting FMT findings and designing future trials. It must be underscored, however, that most the clinical evidence for FMT in CKD remains decidedly preliminary. FMT studies in CKD are either preclinical (rodent models) or small-scale, uncontrolled human case series; the observed correlations between microbial reconstitution and improved renal outcomes do not yet establish causation in the human setting. Findings such as stabilization of renal function or remission of proteinuria derive from small exploratory studies or individual case reports, which may support feasibility and hypothesis generation but cannot establish efficacy. The durability of engraftment, long-term safety, and optimal donor selection, and the generalizability of these findings to broader CKD populations remain open questions that require rigorous investigation in adequately powered RCTs.

**Figure 6:**
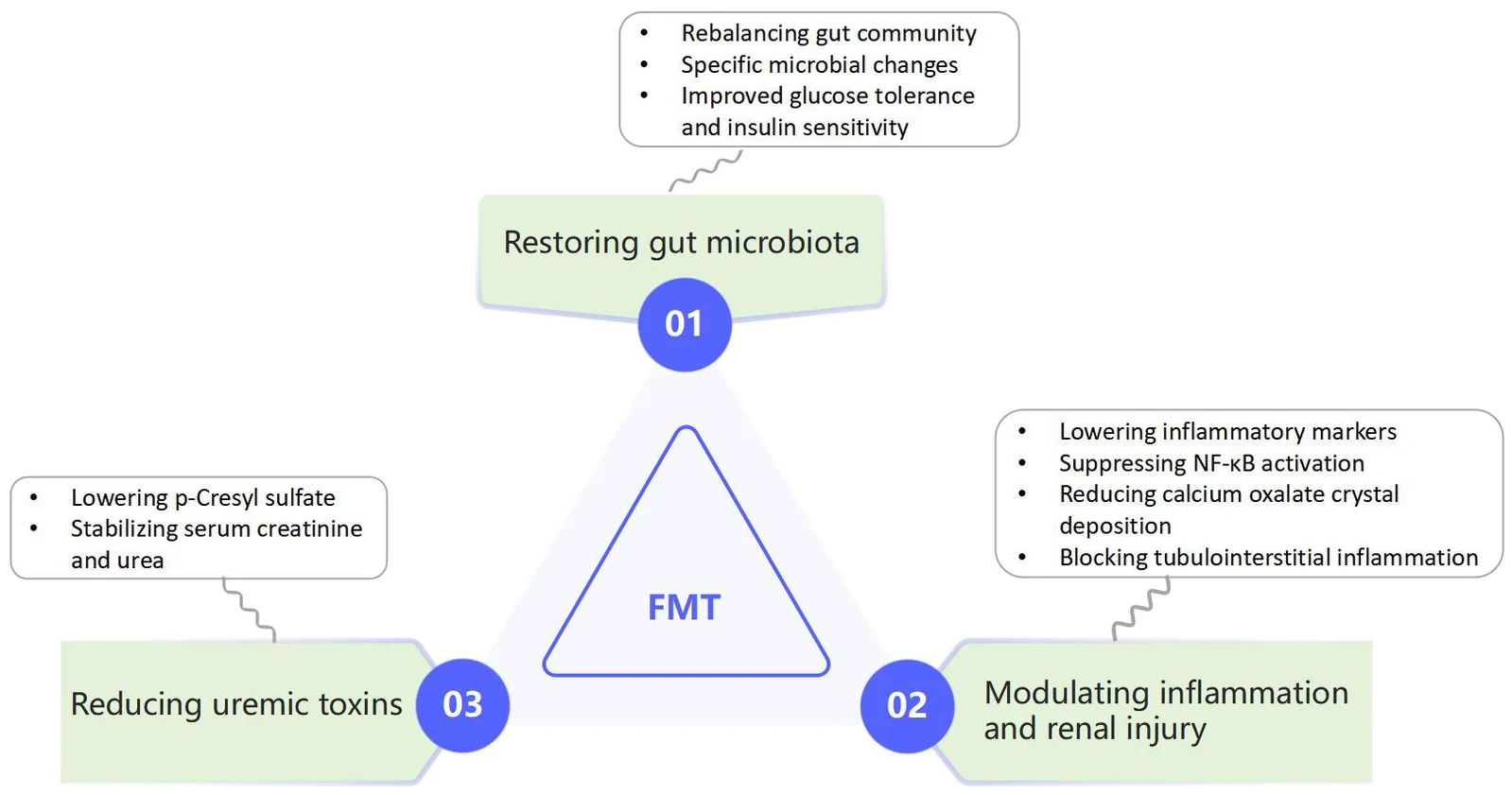
FMT as a promising therapeutic approach for CKD. FMT intervention restored gut microbiota homeostasis by rebalancing microbial composition, which led to improved glucose tolerance and insulin sensitivity. It further reduced uremic toxins, including p-cresyl sulfate, stabilized serum creatinine and urea, and suppressed inflammatory pathways (e.g., NF-κB activation), ultimately mitigating renal injury, calcium oxalate crystal deposition, and tubulointerstitial inflammation.

### 6.1 Preclinical evidence

FMT from healthy donors restores gut eubiosis, normalizes intestinal zonula occludens-1 and SLC26A6 expression, suppresses renal NF-κB activation, and prevents the hyperoxaluria and mineral imbalance that drive calcium-oxalate stone formation (Hunthai et al., 2024). FMT from healthy sham donors restored *Lactobacillus johnsonii* and *Lactobacillus intestinalis*, suppressed microbial tryptophan metabolism and lysine degradation, and thereby halved protein-bound UT accumulation and renal injury in CKD rats (Liu et al., 2022). FMT re-balances the diabetic gut microbiota, enhances glucose tolerance and insulin sensitivity (Iatcu et al., 2021). This metabolic correction slows the progression of diabetic nephropathy and preserves kidney function (Iatcu et al., 2021). FMT reverses diabetic dysbiosis, reduces gut-derived outer-membrane vesicles (OMVs) and their LPS cargo, and consequently blocks caspase-11–mediated tubulointerstitial inflammation and renal injury in diabetic kidney disease (Chen et al., 2023). In a preclinical model of type 2 diabetes mellitus with obesity and diabetic kidney disease, Bastos and colleagues demonstrated that FMT from lean healthy donors attenuated albuminuria, reduced renal mesangial expansion, and mitigated glomerulosclerosis, accompanied by improved glycemic control and reduced systemic inflammation, further substantiating the therapeutic potential of FMT in the specific context of diabetic nephropathy (Bastos et al., 2022). FMT from healthy donors restored *Lactobacillus johnsonii* and *Lactobacillus intestinalis*, suppressed microbial tryptophan/lysine catabolism, and maintained normal renal function and protein-bound UT levels (Liu et al., 2022). In contrast, CKD-donor FMT exacerbated toxin accumulation and kidney damage. FMT from stage-5 CKD patients on a very-low-protein diet successfully engrafted their dysbiotic microbiota, which was marked by an increase in *Escherichia/Shigella/Klebsiella* and a decrease in *Blautia*, into recipient rats (Jiang et al., 2020). This mirrored the donor profiles and induced weight loss without altering renal parameters. Collectively, it mitigated oxalate-induced nephrolithiasis and renal injury. FMT restored microbial diversity, corrected CKD-related dysbiosis, lowered plasma p-cresyl sulfate, and improved glucose tolerance without altering kidney function (Barba et al., 2020).

### 6.2 Clinical evidence (preliminary)

In a small, 6-month trial involving patients with diabetes and/or hypertension and CKD clinical stages 2, 3, and 4, oral FMT halted CKD progression (Arteaga-Muller et al., 2024). Only 13% of FMT-treated patients worsened, versus 54% on placebo, by stabilizing serum creatinine and urea while safely reshaping the gut microbiota to increase Bacteroidetes, Proteobacteria, and Roseburia (Arteaga-Muller et al., 2024). However, the small sample size and short follow-up period preclude definitive conclusions regarding efficacy, and these findings should be considered hypothesis-generating. Corroborating these findings in a broader cohort, Zhong and colleagues conducted a retrospective cohort study in patients with renal dysfunction of various etiologies, demonstrating that washed microbiota transplantation (WMT), a refined FMT procedure, significantly improved renal function as measured by eGFR and serum creatinine, with the therapeutic response being more pronounced in patients with stage 3–4 CKD compared to stage 5, suggesting that earlier intervention may yield greater benefit (Zhong et al., 2023). FMT from healthy donors promptly reduced albuminuria, restored CD89 expression on CD11b^+^ cells, and lowered renal KC chemokine (Lauriero et al., 2021). In contrast, microbiota from progressor IgAN patients increased serum BAFF, galactose-deficient IgA1, and glomerular IgA deposits. This demonstrates that FMT can modulate IgAN severity by re-shaping the gut microbiota (Lauriero et al., 2021). A single FMT reversed chronic diarrhea and improved renal function in a patient with membranous nephropathy, demonstrating the gut-kidney axis as a therapeutic target (Zhou et al., 2021). FMT safely re-balanced the gut microbiota and metabolome in IgA nephropathy patients, reducing 24-hour urinary protein excretion and lowering circulating B-cell counts (Zhi et al., 2024). These effects were linked to reduced renal inflammation and improved clinical outcomes (Zhi et al., 2024). In a complementary case-series report, Zhao and colleagues documented that intensive FMT effectively alleviated refractory IgA nephropathy in two patients who had failed conventional immunosuppressive therapy, with sustained reduction in proteinuria and stabilization of renal function over a 6-month follow-up, providing early evidence that FMT may serve as a salvage therapy for treatment-resistant IgAN (Zhao et al., 2021a). In this first-in-human report, FMT stabilized renal function, drove focal segmental glomerulosclerosis (FSGS) into complete remission (disappearance of proteinuria without glucocorticoid relapse), and corrected dyslipidemia, establishing FMT as a safe adjuvant therapy for FSGS (Zhi et al., 2022). While encouraging, this represents a single observational report and cannot establish efficacy; the findings should be interpreted as hypothesis-generating rather than as evidence of therapeutic effectiveness. Safety data from these preliminary FMT reports were limited, with only mild gastrointestinal discomfort reported; however, larger and longer-term studies are needed to assess serious adverse events in CKD patients.

### 6.3 Indirect evidence from other kidney-related conditions

Beyond studies directly addressing CKD, several investigations have explored FMT in other kidney-related conditions that share pathophysiological features with CKD but are not equivalent. FMT from amoxicillin-treated mice recapitulated the antibiotic’s renal benefits: it expanded stool Alistipes/Odoribacter/Stomatobaculum, depleted Holdemanella/Anaeroplasma, boosted Foxp3^+^CD8^+^ T cells without altering kidney function, significantly reduced post-ischemic fibrosis, and accelerated recovery from acute kidney injury (Gharaie et al., 2023). FMT from healthy donors reversed high-dietary-oxalate hyperoxaluria in rats: it restored Ruminococcaceae_UCG-014 and Parasutterella, enhanced intestinal barrier proteins and oxalate transporters, and thus reduced urinary oxalate excretion and renal calcium oxalate crystal deposition (An et al., 2025). FMT remodeled the gut microbiota, activated a protective Muribaculaceae-Lactobacillus-Bifidobacterium network, and consequently reduced urinary oxalate, calcium, uric acid, creatinine, and urea; decreased serum uric acid and the BUN/creatinine ratio; lessened renal calcium oxalate crystals (from 4+ to 2+); up-regulated osteopontin; and down-regulated renin (Wang et al., 2023c). FMT from hyperuricemic rats transferred dysbiosis and its toxic metabolites, amplifying post-ischemic renal injury, fibrosis, and gut-barrier disruption via NLRP3 inflammasome activation (Zhou et al., 2024). FMT shifts myeloid-derived suppressor cells toward a monocytic phenotype in the blood and kidneys, curbing inflammation and fibrosis after unilateral ureteral obstruction (Wang et al., 2025a). Adoptive transfer of MDSCs replicates these protective effects, identifying MDSCs as key mediators of FMT’s renal benefit (Wang et al., 2025a). It is important to note that these conditions, AKI, hyperoxaluria, calcium oxalate nephrolithiasis, hyperuricemia, and unilateral ureteral obstruction, are pathophysiologically distinct from CKD. Findings from these models provide indirect mechanistic insights into the potential of FMT to modulate renal injury and fibrosis, but they should not be interpreted as direct evidence of efficacy in CKD progression. These studies are discussed separately to acknowledge the broader landscape of FMT research in kidney disease.

## 7 Emerging role of TCM in CKD by regulating gut microecology

TCM has been increasingly recognized for its unique advantages and definite efficacy in treating CKD, with a growing body of evidence demonstrating that TCM interventions, ranging from composite formulae to single herbs and active monomers, can slow disease progression and improve renal function. Recent comprehensive reviews have systematically consolidated these findings, revealing that TCM formulations such as Uremic Clearance Granules, Niaoduqing, and Jian-Pi-Yi-Shen Formula exert renoprotective effects through coordinated modulation of gut microbiota, metabolites, and immune homeostasis, including the rebalancing of Th17/Treg immunity and enrichment of beneficial taxa such as Bifidobacterium animalis (Cai et al., 2025; Yuan et al., 2025; Acquah-Mills et al., 2025; Li and Li, 2025; Wu et al., 2025). Single herb extracts, including anthraquinones from Rheum officinale Baill and other bioactive components, have demonstrated renal fibrosis attenuation through regulation of pro-inflammatory (IκB/NF-κB and Keap1/Nrf2), pro-fibrotic (TGF-β1/Smad and Wnt/β-catenin), and AMPK/PI3K signaling pathways (Feng et al., 2025; Miao et al., 2025; Wang et al., 2023d; Gao et al., 2025). Furthermore, herbal monomers such as emodin, piceatannol, neohesperidin, madecassoside, and isoquercitrin have been shown to ameliorate CKD through microbiota-dependent mechanisms, including the restoration of probiotics like *Lactobacillus johnsonii, Bacteroides ovatus*, and *Bacteroides fragilis*, as well as the suppression of uremic toxin biosynthesis via tryptophan metabolism modulation (Wang et al., 2025b; Wang et al., 2024; Ajibade et al., 2024; Liu et al., 2025b; Ben-Azu et al., 2025). These findings collectively establish a clear mechanistic framework linking TCM interventions to gut microbiota modulation, providing both a foundation for understanding past research and a roadmap for future investigations aimed at translating these microbiota-dependent mechanisms into clinical applications. From this theoretical perspective, TCM views CKD as a condition where root deficiency and branch excess coexist throughout its course. Treatment focuses on boosting *qi*, fortifying the kidneys, activating blood, resolving stasis, clearing heat-dampness, draining turbidity, and reducing edema. These treatment methods are simultaneously multitargeted and multifunctional (Wang et al., 2022b).TCM exerts its anti-CKD effects by regulating the intestinal microecology (**Figure 7**), a concept that is best appreciated when visualizing the concurrent recovery of colonic crypt architecture, restoration of continuous epithelial occludin staining, and regression of renal tubulointerstitial scarring. Nevertheless, the mechanistic interpretations of TCM actions are largely based on compound-gut microbiota interaction studies in rodent models; human pharmacokinetic data for herbal monomers, their bioavailability in the gut lumen, and their direct versus microbiota-mediated effects remain sparse, and the stage-dependent efficacy of TCM in early versus advanced CKD has yet to be systematically investigated.

**Figure 7:**
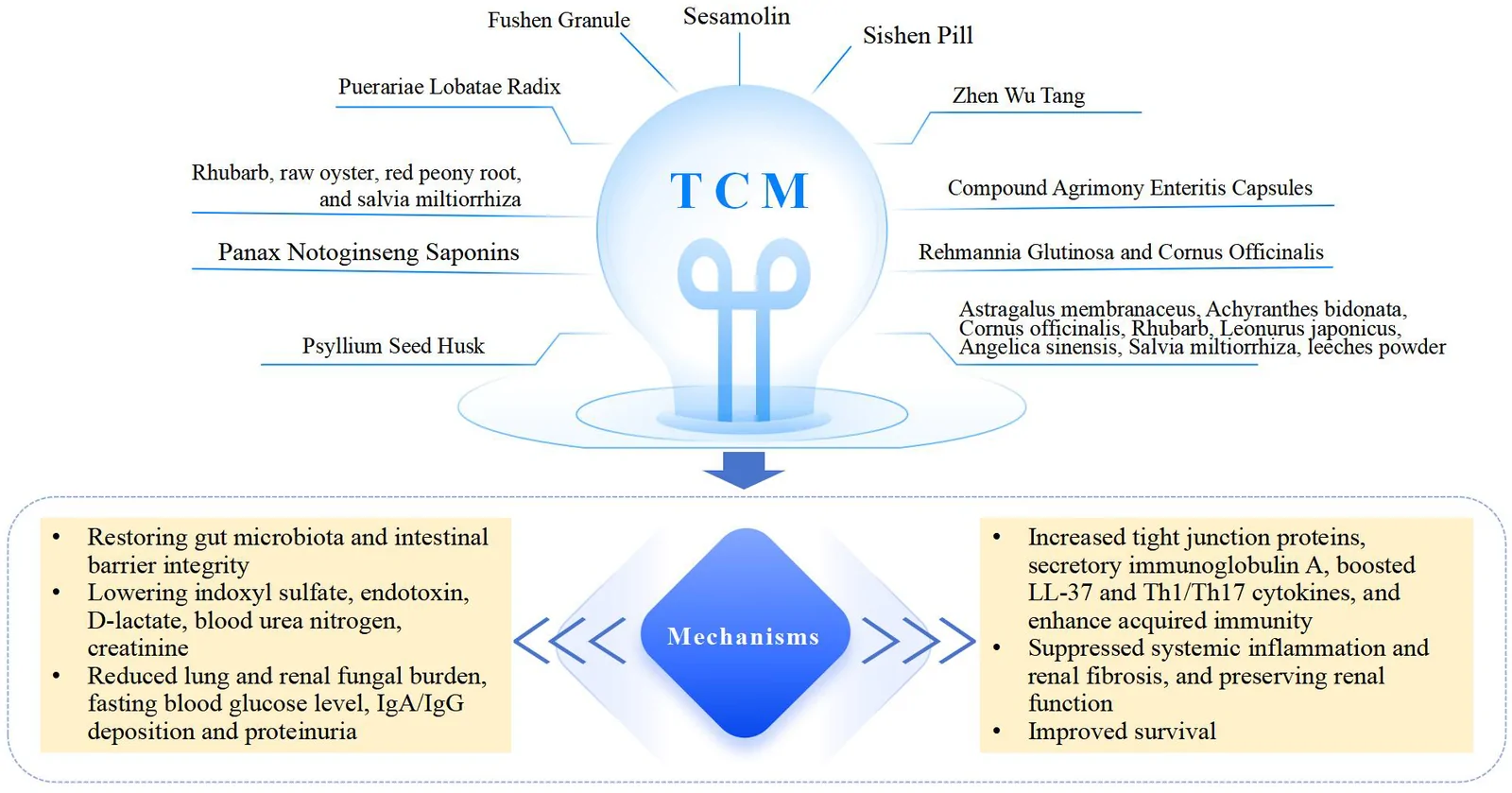
Emerging role of TCM in CKD by regulating gut microecology. TCM alleviates CKD by restoring gut microbiota balance and intestinal barrier integrity, thereby reducing uremic toxins (indoxyl sulfate, D-lactate), systemic inflammation, and renal fibrosis while improving renal function and survival. At the cellular level, these therapeutic actions are represented as reconstitution of the colonic epithelial glycocalyx, reappearance of continuous occludin-positive tight junction belts, and reduction of subepithelial neutrophil infiltration. In the kidney, TCM treatment is iconographically linked to decreased tubular expression of fibronectin and α-SMA, lowered accumulation of immune complexes along the glomerular basement membrane, and diminished proteinaceous casts in tubular lumens. Mechanistically, TCM enhances host immunity by upregulating tight junction proteins, secretory immunoglobulin A, and antimicrobial peptides such as LL-37, alongside modulating Th1/Th17 cytokine responses and reducing immune complex deposition and proteinuria.

### 7.1 Preclinical evidence

Puerariae lobatae Radix rescues high-salt-induced CKD mice by restoring gut eubiosis, enriching Akkermansia and Bifidobacterium, repairing the intestinal barrier, suppressing systemic inflammation, and down-regulating renal Wnt/β-catenin signaling, effects that reduce renal fibrosis and preserve renal function (Wu et al., 2024b). Notably, these therapeutic benefits are transferable via FMT, confirming a microbiota-dependent mechanism underlying the protective actions of Puerariae lobatae Radix (Wu et al., 2024b). Corroborating and extending this mechanistic paradigm, a recent study demonstrated that polyporusterone A, a bioactive steroidal compound, and the Chinese traditional patent medicine ureic clearance granule exert renoprotective effects in unilateral ureteral obstruction (UUO) rats through coordinated gut microbiota modulation and intrarenal signaling suppression (Liu et al., 2026a). Specifically, both interventions significantly increased the abundance of *E. aldenensis* and *L. acidipiscis*, while normalizing dysregulated microbial-derived tryptophan catabolites that are known to activate pathogenic pathways in the kidney. Mechanistically, polyporusterone A and ureic clearance granule suppressed intrarenal aryl hydrocarbon receptor (AhR) signaling and the Wnt1/β-catenin pathway, two interconnected axes that drive renal inflammation and fibrotic progression. These findings not only reinforce the concept that distinct TCM formulations and their active constituents can converge on shared gut-microbiome-host signaling axes, but also identify *E. aldenensis* and *L. acidipiscis* as potential microbial biomarkers for monitoring therapeutic response to TCM-based interventions in CKD. Sishen Pill rescues diarrhea in mice with kidney-yang deficiency syndrome by enriching *Lactobacillus johnsonii*, boosting SCFAs (propionate, butyrate, isobutyrate, isovalerate), lowering serum creatinine, blood urea nitrogen, IL-6, and TNF-α, and increasing colonic secretory immunoglobulin A, thereby restoring gut-kidney homeostasis (Li et al., 2024). Compound Agrimony Enteritis Capsules outperform berberine by restoring intestinal *Bacteroides*, boosting LL-37 and Th1/Th17 cytokines (IFN-γ, TNF-α, IL-17A) while lowering IL-6 and MCP-1. Thereby, it modulates acquired immune responses, reduces lung and renal fungal burden, and significantly improves survival in invasive candidiasis mice (Li et al., 2021). Fushen Granule reduces creatinine, blood urea nitrogen, microalbumin, D-lactate, endotoxin, and intestinal-derived toxins (indophenol and p-cresol sulfate) in CRF rats by restoring *Firmicutes*, suppressing *Bacteroidetes*, and increasing *Lactobacillus*; these benefits were FMT-transferable, confirming a gut-dependent renoprotective mechanism (Wang et al., 2023a). Psyllium seed husk (extracted from the seeds of Plantago ovata) lowers creatinine and blood urea nitrogen, mitigates tubular-interstitial damage, restores gut microbiota and barrier integrity, and suppresses systemic IL-1, IL-6, and indoxyl sulfate, demonstrating its potential to slow CKD progression via the gut-kidney axis (Hu et al., 2023). Li and colleagues revealed that Zhen Wu Tang, (composed of *Aconitum carmichaelii* Debeaux, *Poria cocos* (Schw.) Wolf, *Atractylodes macrocephala* Koidz, *Paeonia lactiflora* Pall, and *Zingiber officinale* Roscoe in a ratio of 3:3:2:3:3, reversed gut dysbiosis in rats with IgA nephropathy, normalized 1,403 perturbed serum metabolites associated with energy and nucleotide metabolism, and significantly reduced renal IgA/IgG deposition and proteinuria, thus protecting kidney function (Li et al., 2020a). These findings underscore that TCM formulae may be more effective when matched to specific CKD subtypes, Zhen Wu Tang being particularly suited to IgAN with prominent yang deficiency patterns, whereas Puerariae lobatae Radix shows greater benefit in hypertensive nephropathy and DKD due to its dual actions on blood pressure regulation and insulin sensitivity, highlighting the importance of syndrome-based differentiation in TCM-guided microbiota modulation. *Rehmannia glutinosa* Libosch and *Cornus officinalis* Sieb herb couple extract normalizes the CKD-distorted gut microbiota (restoring Bacteroidetes and suppressing Firmicutes), repairs the intestinal barrier by up-regulating zonula occludens 1, claudin-1, and occludin-1, and thus attenuates adenine-induced chronic kidney disease in rats (Wang et al., 2022a). Sesamolin, a major lignan in sesame oil from sesame seeds (*Sesamum indicum* L.), can ameliorate high-fat and high-fructose diet-induced kidney injury in mice by restoring intestinal barrier integrity, reshaping the microbiota, and suppressing endotoxin-TLR4-NF-κB–driven inflammation (Yang et al., 2023). Xie et al. found that Panax notoginseng saponins, the main active ingredient of *Panax notoginseng (Burk.) F.H. Chen*, protected against adenine-induced CKD in rats by suppressing renal inflammation/fibrosis, restoring intestinal barrier integrity, and rebalancing gut microbiota and their metabolites (Xie et al., 2022). To facilitate comparison of preclinical findings, we have summarized key TCM and FMT preclinical studies in **Supplementary Table 2**, including the model organism, intervention, mechanistic findings, and translational relevance. This allows to quickly identify the most robust preclinical evidence supporting microbiota-dependent renoprotective mechanisms.

### 7.2 Clinical evidence

The clinical evidence for TCM in CKD is presented below, with each study reporting the formulation, dose, patient population, study design, and key outcomes. High-position colon dialysis combined with TCM (retention enema using a formula consisting of *Rheum officinale* Baill (15 g), *Astragalus membranaceus* Bunge (45 g), *Salvia miltiorrhiza* Bunge (45 g), oyster shell (45 g), *Ligusticum chuanxiong* Hort (25 g), and *Carthamus tinctorius* L. (15 g), significantly improved renal function in patients with CKD stages 4–5 (Deng et al., 2023). This therapeutic approach achieved such effects by reducing levels of indoxyl sulfate, endotoxin, and D-lactate; restoring intestinal barrier integrity (as evidenced by decreased *Escherichia coli* and increased *Bifidobacterium* and *Lactobacillus*); and reshaping the gut microecology, all without causing adverse effects (Deng et al., 2023). The rectal infusion of TCM (Astragalus membranaceus 50 g, Achyranthes bidentata 10 g, Cornus officinalis 10 g, Rhubarb 10 g, Leonurus japonicus 10 g, Angelica sinensis 10 g, Salvia miltiorrhiza 10 g, leeches powder 3 g, 100 mL each time after decoction, twice a day) combined with Maslow’s hierarchy of needs theory for nursing, compared with TCM treatment alone, further reduces the levels of urea nitrogen, serum creatinine, fasting blood glucose, and 2-hour postprandial blood glucose, as well as the scores on the Hamilton Anxiety Scale and the Hamilton Depression Scale (Li et al., 2025). It also decreases the levels of Bacteroidetes and Actinomycetes, but increases the levels of Firmicutes (Li et al., 2025). For patients with stage 3 to 4 CKD characterized by dampness, turbidity, and blood stasis, compared with conventional colonic dialysis treatment, conventional colonic dialysis combined with the enema formula for renal failure (150 ml of concentrated decoction of rhubarb, raw oyster, red peony root, and salvia miltiorrhiza, once every other day, for a course of 4 weeks) further reduces the levels of indoxyl sulfate, serum creatinine, urea nitrogen, serum endotoxin, D-lactic acid, and *Escherichia coli* (Zhang et al., 2023b). The eGFR is further increased in the combination group (P < 0.05), but neither treatment had a significant effect on *Bifidobacterium* (Zhang et al., 2023b).

## 8 Discussion

Over the past decade, the gut-kidney axis has emerged as a central pathophysiological pillar of CKD, with CKD progression to ESKD closely linked to accumulation of toxic metabolites driven by gut microbial dysbiosis, impaired renal clearance, intestinal barrier dysfunction, and systemic inflammation (Giordano et al., 2021). These findings offer new therapeutic possibilities for managing the progression of kidney diseases and preventing adverse consequences in CKD patients. The accelerating pace of recent publications has further refined these mechanistic insights and progressively expanded the clinical evidence base, particularly through well-designed interventional studies that evaluate microbiota-targeted therapies across diverse CKD populations.

By integrating preclinical and clinical studies, this review provides a panoramic map of how microbiome-directed interventions can be utilized against renal decline. However, it is essential to recognize the hierarchical nature of the evidence presented: the most robust conclusions derive from network meta-analyses of RCTs, followed by individual RCTs, then observational studies, with case reports and preclinical animal experiments providing mechanistic hypotheses rather than definitive clinical proof. Throughout this review, we have deliberately separated preclinical from clinical evidence and have indicated the study design and CKD stage for each cited clinical study. Moreover, findings involving changes in microbiota composition or inflammatory markers are presented as supportive mechanistic or correlative observations; they are not interpreted as direct evidence that an intervention slows CKD progression unless renal outcomes (eGFR, serum creatinine, proteinuria, or hard clinical endpoints) were directly assessed. Three overarching insights emerge: compositional dysbiosis of the microbiome is not merely a biomarker but an active driver of CKD progression; precision microecological formulations and FMT reproducibly attenuate UT and systemic inflammation, resulting in measurable renoprotection; and emerging TCM operates, at least in part, through a microbiota-dependent epigenetic-metabolic circuitry. Critically, each of these pathways can be visualized through distinct pathological hallmarks: disruption of intestinal tight junction strands, translocation of endotoxin-laden vesicles into the lamina propria, oxidative injury to proximal tubular mitochondria, and progressive interstitial fibrosis with α-SMA-positive myofibroblasts. Responses to probiotic/prebiotic therapies vary widely based on CKD severity and disease type. Early non-dialysis CKD responds well to gut microbe regulation, while advanced non-dialysis, hemodialysis and peritoneal CKD show weak or inconsistent toxin-lowering effects due to severe gut dysbiosis and dialysis-related complications. Different kidney diseases have unique gut flora and pathogenesis, so microbial treatments produce disparate effects and research must classify patients by both CKD stage and etiology instead of drawing universal conclusions.

CKD and gut microbiota engage in a vicious cycle: Dysbiosis, marked by depleted Lactobacillaceae/Prevotellaceae and overgrown Enterobacteria/Enterococci, is associated with accelerated fermentation of tyrosine and tryptophan into UT (indoxyl sulfate, p-cresyl sulfate, trimethylamine N-oxide) (Al Khodor and Shatat, 2017). These UT accumulate as GFR declines, while the retained toxins may further distort the microbial composition and fuel systemic inflammation. Jointly, they are thought to propel CKD progression and raise mortality (Al Khodor and Shatat, 2017). A network meta-analysis of randomized clinical trials showed that probiotics lowered serum triglycerides, while probiotics and synbiotics improved systemic oxidative stress by decreasing MDA and increasing glutathione or total antioxidant capacity (Tan et al., 2022). Recent comprehensive reviews have extended these findings by elucidating the underlying molecular pathways: CKD-associated gut dysbiosis impairs intestinal epithelial barrier function, reducing beneficial taxa such as *Lactobacillus reuteri* and *Bifidobacterium animalis* while increasing pathogens including *Eggerthella lenta* and *Fusobacterium nucleatum*, with consequent accumulation of microbial-derived metabolites including IAA, TMAO, and indoxyl sulfate that drive renal inflammation and fibrosis. Importantly, probiotics such as *Parabacteroides goldsteinii, Lactobacillus johnsonii, Bacteroides ovatus*, and *Bacteroides fragilis* have shown renoprotective effects through the regulation of FXR, GLP-1R, SGLT2, and Nrf2 pathways via metabolites including IAld, hyodeoxycholic acid, and 1,5-anhydroglucitol. The clinical translation of these mechanistic insights is supported by evidence across different CKD stages, with probiotics and/or synbiotics demonstrating improvements in creatinine, uremic parameters, and gastrointestinal symptoms, alongside modulation of gut microbiota and reduction of serum endotoxin. Notably, the *Lactobacillus johnsonii*-IAld-AhR axis represents one of the most compelling strain-specific mechanisms identified to date, offering a clear pathophysiological rationale for targeted probiotic selection in CKD management. The relationship between kidney disease and the gut microbiota can be summarized as follows: during the onset and progression of CKD, the gut microbial ecosystem is disrupted (Ondrussek-Sekac et al., 2021). This disruption is characterized by altered microbial composition, increased production of gut-derived UT, impaired intestinal barrier function, and elevated endotoxin levels (Ondrussek-Sekac et al., 2021). These associations, however, do not prove causation; the observed correlations between specific microbial shifts and renal functional decline may reflect either direct pathogenicity, secondary responses to the uremic milieu, or confounding by shared risk factors such as diet, medication, and comorbidities. Western medicine commonly employs microbiota-targeted interventions such as probiotics, prebiotics, synbiotics, and FMT to rebalance the gut microbiota, attenuate inflammation, restore mucosal integrity, and reduce endotoxemia. Although current CKD therapies remain suboptimal and their mechanisms are not fully elucidated, microbiota-based treatments have shown promise in improving renal function, reducing toxin accumulation, alleviating the inflammatory status, correcting dysbiosis, and relieving gastrointestinal symptoms, representing a potentially valuable approach for CKD management. Critically, the clinical evidence for FMT in CKD remains at an early stage and is further constrained by stage-dependent and subtype-specific considerations. The available human data consist largely of small exploratory trials, case series, and individual case reports, which are insufficient to establish efficacy or safety. Within this limited evidence base, the few positive signals such as stabilization of renal function in stage 2–4 CKD, proteinuria reduction in IgAN, and remission in FSGS, derive from distinct patient populations with different underlying pathophysiologies. These findings should not be generalized across CKD subtypes or stages without explicit qualification. The therapeutic potential of FMT in early versus advanced CKD, in diabetic versus non-diabetic etiologies, and in IgAN versus FSGS versus other subtypes, remains largely unexplored and requires dedicated investigation in adequately powered, subtype-stratified trials.

Three overarching limitations, noted throughout this review, warrant reiteration: mechanistic insights are predominantly derived from rodent models and require human validation; most reported microbial associations remain correlative rather than causally proven; and therapeutic responsiveness exhibits marked stage-dependent heterogeneity, with more consistent benefits in early-stage non-dialysis CKD and more variable responses in advanced disease and dialysis populations.

Our synthesis extends prior studies by disaggregating the effects of single-strain probiotics, multi-strain consortia, prebiotics, synbiotics, and FMT across the entire CKD continuum (Yang et al., 2021; Cedillo-Flores et al., 2025). Network meta-analyses suggest differential efficacy of gut microbiota modulators: prebiotics may be preferred for reducing UT and certain inflammatory markers (IL-6, TNF-α, hs-CRP); synbiotics for endotoxin and CRP reduction; and probiotics for lowering serum creatinine in CKD (Yu et al., 2022; Tan et al., 2022). However, several critical caveats must be acknowledged when interpreting these rankings. First, these findings derive from indirect comparisons rather than direct head-to-head trials; the number of studies per comparison is small, and substantial heterogeneity exists in probiotic strains, prebiotic types, dosages, treatment durations, and patient populations. Second, another network meta-analysis found no significant effect of any intervention on uremic toxins, highlighting the inconsistency across meta-analyses and the need for cautious interpretation. Third, the certainty of evidence for most comparisons is low to moderate, largely due to imprecision, inconsistency, and indirectness of the evidence base. Therefore, the rankings should be considered as hypothesis-generating signals that require validation in well-powered, head-to-head RCTs, rather than as firm evidence of therapeutic superiority. Compared with the healthy control group, differences in the composition of the gut microbiota were observed in preclinical animal models and patients with type 2 diabetes accompanied by diabetic nephropathy (Iatcu et al., 2021). Microecological dysregulation leads to cellular inflammation and tubulointerstitial damage (Chen et al., 2023). FMT alters the abundance of the microbiota and delays the progression of CKD, which is associated with the down-regulation of proinflammatory cytokines and the attenuation of renal fibrosis (Arteaga-Muller et al., 2024; Wang et al., 2025a). Preliminary clinical reports have suggested potential stabilization of renal function in small patient cohorts, but these observations require confirmation in larger, well-controlled trials. Evidence from other kidney-related conditions, including AKI, hyperoxaluria, nephrolithiasis, hyperuricemia, and unilateral ureteral obstruction, provides indirect mechanistic insights but should not be conflated with direct evidence of FMT efficacy in CKD progression. The therapeutic potential of TCM has long been recognized and continues to attract global attention in treating human diseases (Zhu et al., 2021b; Zhu et al., 2022; Xiang et al., 2021). TCM formulae such as Zhen Wu Tang and Puerariae lobatae Radix offer a notable example of multi-omics integration, where botanical xenobiotics function as prebiotic-like “ecological engineers”.

However, not all microecological preparations can improve renal function or UT. This may be related to the different functions of microecological preparations. A further nuance concerns the differential emphasis on specific gut-derived metabolites across the current literature: while TMAO has attracted considerable attention due to its cardiovascular risk association and established link to dietary phosphatidylcholine, the mechanistic roles of SCFAs and bile acids in CKD are equally, if not more, consequential. SCFAs serve as primary anti-inflammatory effectors, whereas bile acids act as pleiotropic metabolic integrators, with direct implications for renal energy homeostasis, fibrosis, and immune regulation. The relative neglect of these pathways in clinical interventional studies represents a critical gap; future investigations should adopt a more balanced metabolomic perspective that simultaneously quantifies uremic toxins, SCFAs, and bile acid species to capture the full spectrum of gut-kidney metabolic crosstalk. β-Glucan prebiotic had no effect on kidney function: serum urea, creatinine, and eGFR remained unchanged over 14 weeks (Ebrahim et al., 2022). Across the entire cohort, 21 g/day of unripe banana flour failed to lower serum indoxyl sulfate, p-cresyl sulfate, or indole-3-acetic acid. Although, in a compliant subgroup, there was a significant 21% reduction in indoxyl sulfate (de Andrade et al., 2021). 60 days of synbiotics (Lactobacillus acidophilus and Bifidobacterium longum 5x10^9^ CFU) did not alter serum indoxyl sulfate levels (p = 0.438) in chronic hemodialysis patients with gastrointestinal complaints, difficulty defecating, feces with hard consistency, or a bowel movement frequency of fewer than three times per week (Lydia et al., 2022). Another network meta-analysis of randomized clinical trials showed that none of the interventions altered UT in patients with CKD (Tan et al., 2022). More high-quality research is needed to verify its exact efficacy and the best usage plan.

There are still many deficiencies in the current research advances. First, only a few clinical studies have confirmed the above-mentioned efficacy. This may be related to the insufficient dosage of microecological preparations, small sample size, short intervention time, large individual differences among patients, and the nature of the intestinal flora itself. Second, the application of many new types of microecological preparations is currently limited to animal experiments. Third, there is significant heterogeneity in the strains, dosages, and intervention protocols used among different studies, which limits the comparability and generalizability of the results. Fourth, the majority of clinical trials are short (<less than 12 weeks) and lack the power to evaluate hard renal outcomes. Fifth, safety data for FMT in immunocompromised CKD patients are limited; the durability of microbiome engraftment beyond 6 months is largely unknown. Additionally, the field lacks precision frameworks similar to oncology’s “tumor-agnostic” microbiome therapeutics. Patient stratification based on baseline enterotypes or genetic polymorphisms in xenobiotic-metabolizing enzymes remains exploratory. Specifically with respect to the network meta-analyses cited in this review, we acknowledge the following limitations: (i) the small number of trials per intervention category limits the precision of estimates; (ii) substantial clinical heterogeneity (variations in CKD stage, dialysis status, probiotic strains, prebiotic types, doses, and treatment durations) reduces the generalizability of findings; (iii) the reliance on indirect comparisons introduces uncertainty, as no large head-to-head trials have directly compared probiotics, prebiotics, and synbiotics within the same CKD population; and (iv) some meta-analyses have yielded discordant conclusions regarding efficacy on uremic toxins and renal function. These limitations preclude definitive conclusions regarding the superiority of any single intervention category. While the therapeutic potential of microbiota-targeted interventions in CKD is promising, their safety in medically vulnerable patients must be rigorously evaluated. Future trials should systematically collect and report adverse events, establish standardized donor screening and product quality protocols for FMT and TCM, and conduct long-term safety surveillance to inform evidence-based clinical recommendations.

The gut microbiota plays a key role in the pathogenesis and progression of CKD. Microecological preparations, fecal microbiota transplantation, and TCM all show potential in regulating intestinal microecology and improving renal function. Although there is already a large amount of basic research and preliminary clinical evidence to support these intervention methods, more high-quality studies are still needed to verify their long-term efficacy and safety and to explore individualized treatment pathways. Future research should focus on mechanism analysis, the establishment of standardized operation procedures, and the integration of interdisciplinary technologies to accelerate the clinical translation of these findings.

Promoting international collaboration and establishing a unified microbiota intervention standard and evaluation system are of vital importance for enhancing research quality. At the same time, public education and policy support should be strengthened to promote the standardized application of microecological therapy in CKD management. In the future, under the premise of ensuring safety, larger-scale high-quality clinical trials still need to be carried out to study the effectiveness of microecological preparations. In addition, it is necessary to explore new experimental techniques to understand more about the functions of the intestinal flora and provide more scientific basis for clinical applications. Future research should focus on individualized microbiota intervention strategies, including precise microbiota regulation based on metagenomics and metabolomics, syndrome differentiation and treatment for different TCM syndrome types, and the development of microbiota prediction models combined with artificial intelligence.

To transcend these limitations, we propose a roadmap anchored in causal inference and precision medicine. Longitudinal, multi-center trials with adaptive Bayesian designs are needed to compare head-to-head synbiotic cocktails, autologous versus allogenic FMT, and TCM-derived microbiota consortia across CKD stages 2-4. End-points must include composite renal failure events, cardiovascular mortality, and quality-adjusted life years. Integration of host genome–microbiome–metabolome multi-omics will enable machine-learning models to predict responders and refine exclusion criteria (e.g., Enterobacteriaceae dominance or high baseline indoxyl sulfate). Development of next-generation live biotherapeutic products that incorporate CRISPR-edited butyrate super-producers or indole-degrading strains could circumvent engraftment failure and yield scalable, GMP-grade alternatives to donor-derived FMT.

Finally, regulatory science must evolve: the FDA and EMA should establish harmonized safety and potency assays for microbiota therapeutics, akin to those governing monoclonal antibodies. The convergence of microbiome science, systems biology, and integrative nephrology has positioned the gut-kidney axis as a tractable therapeutic frontier. Whether this promise translates into a meaningful delay of dialysis for millions will depend on rigorously designed, mechanism-anchored trials that embrace precision medicine principles and regulatory innovation.

Beyond the specific limitations noted above regarding animal-to-human translation, causal inference, and stage-dependent heterogeneity, several additional constraints affect the current evidence base. First, the vast majority of clinical trials are of short duration (<12 weeks) and lack sufficient power to evaluate hard renal endpoints; surrogate markers including eGFR and uremic toxin levels, while informative, do not always correlate with long-term clinical outcomes. Second, the marked heterogeneity in probiotic strains, prebiotic types, dosages, and synbiotic combinations across studies precludes direct comparison and meta-analytic pooling, limiting the generalizability of conclusions. Third, safety data for FMT in immunocompromized CKD and transplant populations remain scarce, with risks of infection, dysbiosis transmission, and long-term immunological consequences largely unexplored. Fourth, the durability of microbiota engraftment after intervention cessation is unknown, and most studies lack longitudinal follow-up beyond the intervention period. Future research should prioritize: (i) large-scale, multicenter clinical trials with adaptive designs that stratify participants by CKD stage and baseline enterotype; (ii) integration of multi-omics with machine-learning approaches to identify predictive biomarkers of therapeutic response; (iii) head-to-head comparisons of different intervention modalities within the same population; (iv) standardized reporting frameworks for microbiota therapeutics to enhance cross-study comparability; and (v) long-term safety registries for FMT and next-generation live biotherapeutic products in CKD patients.

## 9 Conclusion

Despite major existing limitations relying heavily on rodent mechanistic research, mostly correlational human data, inconsistent study designs, and variable treatment responses across CKD stages, the bidirectional gut-kidney axis is confirmed as a key driver of CKD progression. Microbe-targeted therapies share unified underlying mechanisms and show promising translational prospects, yet critical unknowns remain. Network meta-analysis results comparing their efficacy carry low-to-moderate evidence confidence due to indirect cross-study comparisons and conflicting data on uremic toxin reduction, mandating direct head-to-head human trials to identify superior regimens. FMT’s theoretical benefits are strong with tentative positive early observations, but human clinical evidence is far too sparse to support routine clinical use. To unlock this therapeutic potential, standardized microbiome testing, mechanism-focused clinical trial frameworks, and dedicated regulatory rules for microbial biologics are urgently needed. All future trials must pair efficacy assessments with strict long-term safety monitoring to protect vulnerable CKD patients from infectious or organ toxic risks. Expanding RCTs and real-world cohort research will eventually clarify clinical guidelines and translate gut-kidney research into tangible improvements for people living with CKD.
