# Supplementary material for: Microbiota-targeted interventions in chronic kidney disease: probiotics, prebiotics, synbiotics, fecal microbiota transplantation, and traditional Chinese medicine
Source: Front Cell Infect Microbiol. 2026 Oct 13;16:1900027. doi: 10.3389/fcimb.2026.1900027 (PMC13741479; doi:10.3389/fcimb.2026.1900027)
Supplement: Supplementary file 1 [file DataSheet1.docx]

**Supplementary Table 1. Summary of clinical evidence for microbiota-targeted interventions in CKD.**

| **Intervention/Ref.** | **Study design** | **CKD stage / dialysis Status** | **Intervention (dose)** | **Duration** | **Main renal & metabolic outcomes** | **Major limitations** |
| --- | --- | --- | --- | --- | --- | --- |
| **Probiotics** |  |  |  |  |  |  |
| Simeoni et al., 2019 | Randomized, open-label, placebo-controlled | Stage 3a (non-dialysis) | L. acidophilus, L. reuteri, B. bifidum, S. thermophilus (probiotic formulation) | 2 months | ↓ Urinary indican & 3-methylindole; ↓ CRP; restored fecal Lactobacillales & Bifidobacteria | Small sample; open-label design |
| De Mauri et al., 2022 | Randomized, placebo-controlled | Advanced CKD (non-dialysis) | B. longum + L. reuteri (with low-protein diet) | 6 months | ↓ Indoxyl sulfate rise; ↓ pro-atherogenic toxins | Small sample; combined intervention (diet + probiotic) |
| Liu et al., 2020 | Randomized trial | Hemodialysis | Probiotic formulation | 3 months | ↓ Indole-3-acetic acid-O-glucuronide; ↑ Bacteroidaceae & Enterococcaceae | **Small sample** |
| Choi et al., 2022 | RCT | Hemodialysis | B. bifidum BGN4 + B. longum BORI (2×10¹⁰ CFU b.i.d.) | 3 months | ↓ Serum calprotectin & LPS-stimulated IL-6; ↑ CD4⁺CD25⁺ Tregs; ↑ SCFA production | Small sample |
| Tungsanga et al., 2022 | RCT (preclinical-to-clinical translation) | 5/6 nephrectomy mouse model | L. rhamnosus L34 (daily) | 14 weeks | ↓ Serum endotoxin (P=0.03); ↓ TNF-α (P=0.01); ↓ kidney fibrosis (P<0.01); ↓ serum creatinine & proteinuria | Preclinical model; translation to humans pending |
| **Prebiotics** |  |  |  |  |  |  |
| Esgalhado et al., 2020 | Randomized, double-blind, crossover, placebo-controlled | Hemodialysis | Resistant starch type-2 enriched cookies (16 g/day) | 4 weeks | ↓ Plasma indoxyl sulfate; ↑ Nrf2 mRNA & NQO1 (antioxidant response); no change in NF-κB | Short duration; small sample |
| Kemp et al., 2021 | Randomized controlled trial | Hemodialysis | RS2-enriched cookies (16 g/day) | 4 weeks | ↑ Roseburia & Ruminococcus gauvreauii (SCFA producers); ↓ Dialister (pro-inflammatory) | Short duration |
| Li et al., 2020b | Randomized crossover trial | Peritoneal dialysis | Inulin-type fructans (10 g/day) | 3 months | ↓ Fecal indole (-10.1 vs +13.3 μg/g; P=0.040); ↓ Bacteroides thetaiotaomicron; no change in serum IS or pCS | Small sample |
| He et al., 2022 | Randomized crossover trial | Peritoneal dialysis | Inulin-type fructans (10 g/day) | 3 months | ↓ Serum uric acid (~10%); ↑ fecal UA degradation; ↑ purine-catabolizing Clostridium | Short follow-up |
| Xiong et al., 2023 | Randomized crossover trial | Peritoneal dialysis | Inulin-type fructans (10 g/day) | 3 months | ↑ Firmicutes/Bacteroidetes ratio (P=0.049) | Limited generalizability |
| Ebrahim et al., 2022 | RCT | Stage 3-5 (non-dialysis) | β-Glucan prebiotic | 14 weeks | ↓ Free IS at 8 wks (P=0.003) & 14 wks (P<0.001); ↓ free pCS at 14 wks (P=0.006); NO change in eGFR, creatinine, or urea | No renal function improvement |
| de Andrade et al., 2021 | Randomized, double-blind, placebo-controlled, crossover | Peritoneal dialysis | Unripe banana flour (21 g/day) | 4 weeks | No change in serum IS, pCS, or IAA overall; 21% ↓ IS in compliant subgroup | Short duration; negative primary outcome |
| **Synbiotics** |  |  |  |  |  |  |
| Mitrović et al., 2023 | RCT | Non-dialysis CKD | L. acidophilus, L. casei, B. lactis (32 billion CFU/day + 3.2 g inulin) | 12 weeks | ↑ eGFR (+12%); ↓ IS (-21.5%, P<0.001); ↓ hs-CRP (-39.5%, P<0.001); no change in pCS or TMAO | pCS unchanged |
| Rossi et al., 2016 | RCT (SYNERGY trial) | Predialysis (eGFR 10-30 mL/min/1.73m²) | Synbiotic formulation | 6 weeks | ↓ Serum pCS; ↑ Bifidobacterium; no change in serum IS | Short duration |
| Haghighat et al., 2020 | RCT | Hemodialysis | Synbiotic (probiotics + prebiotics) | 12 weeks | ↓ Serum endotoxin (P=0.036); ↓ hs-CRP & IL-6 (P<0.001); ↓ anti-HSP70 (P=0.002) | No renal functional outcomes reported |
| Lydia et al., 2022 | RCT | Hemodialysis | L. acidophilus + B. longum (5×10⁹ CFU) + prebiotic | 60 days | ↓ Constipation symptoms (P=0.006); ↑ QoL (P=0.001); NO change in serum IS (P=0.438) | Negative biomarker outcome |
| **FMT** |  |  |  |  |  |  |
| Arteaga-Muller et al., 2024 | RCT (exploratory) | Stage 2-4 (diabetes/hypertension) | Oral FMT capsules (healthy donors) | 6 months | Only 13% worsened vs 54% placebo; stabilized creatinine & urea; ↑ Bacteroidetes, Proteobacteria, Roseburia | Small sample |
| Zhi et al., 2024 | Exploratory clinical trial | IgA nephropathy | FMT capsules (healthy donors) | 6 months | ↓ 24-hour proteinuria; ↓ circulating B-cell counts; reshaped gut microbiota & metabolome | Uncontrolled |
| Lauriero et al., 2021 | Humanized mouse model (clinical translation) | IgA nephropathy | FMT from healthy donors | N/A (in vitro) | ↓ Albuminuria; ↑ CD89 expression; ↓ renal KC chemokine; reduced galactose-deficient IgA1 | No human intervention data |
| Zhi et al., 2022 | Case report | FSGS | FMT capsules | 6 months | Complete remission (proteinuria disappearance); stabilized renal function; corrected dyslipidemia | Single case report |
| Zhou et al., 2021 | Case report | Membranous nephropathy | Single FMT | Single dose | Reversed chronic diarrhea; improved renal function | Single case report |
| **TCM** |  |  |  |  |  |  |
| Deng et al., 2023 | Real-world cohort | Stage 4-5 | High-position colon dialysis + TCM retention enema (formula: rhubarb 15g, astragalus 45g, salvia 45g, oyster shell 45g, chuanxiong 25g, safflower 15g) | 4 weeks | ↓ IS, endotoxin, D-lactate; ↑ Bifidobacterium & Lactobacillus; ↓ E. coli; improved renal function | Uncontrolled |
| Zhang et al., 2023b | RCT | Stage 3-4 | Colon dialysis + TCM enema (rhubarb, raw oyster, red peony root, salvia miltiorrhiza; 150 mL every other day) | 4 weeks | ↓ IS, creatinine, BUN, endotoxin, D-lactic acid, E. coli; ↑ eGFR (P<0.05); no change in Bifidobacterium | Short duration |
| Li et al., 2025 | RCT | Diabetic nephropathy | Rectal TCM infusion (astragalus 50g, achyranthes 10g, cornu 10g, rhubarb 10g, leonurus 10g, angelica 10g, salvia 10g, leech powder 3g) + Maslow nursing | Not specified | ↓ BUN, creatinine, FBG, 2h-PBG; ↓ Bacteroidetes & Actinomycetes; ↑ Firmicutes; ↓ HAMA & HAMD scores | No direct renal outcome measure |

Abbreviations: BUN, blood urea nitrogen; CKD, chronic kidney disease; CRP, C-reactive protein; eGFR, estimated glomerular filtration rate; FBG, fasting blood glucose; FMT, fecal microbiota transplantation; FSGS, focal segmental glomerulosclerosis; GI, gastrointestinal; HAMA, Hamilton Anxiety Scale; HAMD, Hamilton Depression Scale; hs-CRP, high-sensitivity C-reactive protein; IAA, indole-3-acetic acid; IgAN, IgA nephropathy; IL-6, interleukin-6; IS, indoxyl sulfate; LPS, lipopolysaccharide; pCS, p-cresyl sulfate; QoL, quality of life; RCT, randomized controlled trial; RS2, resistant starch type-2; SCFA, short-chain fatty acid; TCM, traditional Chinese medicine; TMAO, trimethylamine N-oxide; TNF-α, tumor necrosis factor-alpha; UA, uric acid. Note: FMT case reports and exploratory trials are clearly distinguished from randomized studies; findings are presented as hypothesis-generating unless confirmed by adequately powered RCTs.
