# Supplementary material for: Microbiota-targeted interventions in chronic kidney disease: probiotics, prebiotics, synbiotics, fecal microbiota transplantation, and traditional Chinese medicine
Source: Front Cell Infect Microbiol. 2026 Oct 13;16:1900027. doi: 10.3389/fcimb.2026.1900027 (PMC13741479; doi:10.3389/fcimb.2026.1900027)
Supplement: Supplementary file 2 [file DataSheet2.docx]

**Supplementary Table 2. Key preclinical evidence for TCM and FMT interventions in CKD models.**

| **Intervention** | **Model organism** | **CKD model** | **Key mechanistic findings** | **Translational relevance** | **Reference** |
| --- | --- | --- | --- | --- | --- |
| **TCM formulations & herbal extracts** |  |  |  |  |  |
| Puerariae lobatae Radix | Mouse | High-salt-induced CKD | ↑ Akkermansia & Bifidobacterium; repaired intestinal barrier; ↓ Wnt/β-catenin signaling; ↓ renal fibrosis; FMT-transferable protection | Microbiota-dependent mechanism; potential for hypertensive nephropathy & DKD | Wu et al., 2024b |
| Polyporusterone A + Uremic Clearance Granule | Rat | UUO | ↑ E. aldenensis & L. acidipiscis; normalized tryptophan catabolites; ↓ AhR & Wnt1/β-catenin pathways | Identified microbial biomarkers for TCM response; dual intervention validates shared mechanism | Liu et al., 2026a |
| Sishen Pill | Mouse | Kidney-yang deficiency syndrome with diarrhea | ↑ L. johnsonii; ↑ SCFAs (propionate, butyrate, isobutyrate, isovalerate); ↓ creatinine, BUN, IL-6, TNF-α; ↑ colonic sIgA | Restores gut-kidney homeostasis; applicable to CKD with GI symptoms | Li et al., 2024b |
| Fushen Granule | Rat | CRF | Restored Firmicutes/Bacteroidetes ratio; ↑ Lactobacillus; ↓ creatinine, BUN, microalbumin, D-lactate, endotoxin, IS, pCS; FMT-transferable | Gut-dependent renoprotective mechanism; FMT confirms causality | Wang et al., 2023a |
| Psyllium seed husk | Rat | 5/6 nephrectomy | ↓ Creatinine, BUN; mitigated tubular-interstitial damage; restored gut barrier; ↓ IL-1, IL-6, IS | Potential prebiotic-like effect; clinically available dietary fiber | Hu et al., 2023 |
| Zhen Wu Tang | Rat | IgA nephropathy | Reversed gut dysbiosis; normalized 1,403 perturbed serum metabolites; ↓ renal IgA/IgG deposition & proteinuria | Subtype-specific efficacy (IgAN with yang deficiency); multi-omics integration | Li et al., 2020a |
| Rehmannia-Cornus herb couple | Rat | Adenine-induced CKD | Restored Bacteroidetes/Firmicutes balance; ↑ ZO-1, claudin-1, occludin-1; ↓ creatinine, BUN | Intestinal barrier repair as primary mechanism | Wang et al., 2022a |
| Sesamolin | Mouse | High-fat/high-fructose diet | Restored intestinal barrier; reshaped microbiota; suppressed endotoxin-TLR4-NF-κB inflammation | Dual benefit for metabolic CKD and gut health | Yang et al., 2023 |
| Panax notoginseng saponins | Rat | Adenine-induced CKD | Suppressed renal inflammation/fibrosis; restored intestinal barrier; rebalanced gut microbiota & metabolites | Clinically available herbal extract; multi-target effects | Xie et al., 2022 |
| **FMT studies** |  |  |  |  |  |
| Healthy donor FMT | Rat | CKD (5/6 nephrectomy) | Restored L. johnsonii & L. intestinalis; ↓ tryptophan/lysine catabolism; halved protein-bound UT accumulation; preserved renal function | Demonstrates FMT as causal therapeutic; identifies key protective taxa | Liu et al., 2022 |
| Healthy donor FMT | Mouse | Diabetic kidney disease | Reversed diabetic dysbiosis; ↓ gut-derived OMVs & LPS; blocked caspase-11-mediated tubulointerstitial inflammation | Mechanistic link between FMT, OMVs, and renal inflammation | Chen et al., 2023 |
| CKD-donor FMT | Rat | CKD stage 5 (very-low-protein diet) | Engrafted dysbiotic microbiota (↑ Escherichia/Shigella/Klebsiella; ↓ Blautia); induced weight loss but no renal parameter change | Demonstrates transmissibility of dysbiosis; host factors may limit transfer | Jiang et al., 2020 |
| Healthy donor FMT | Rat | Hyperoxaluria/calcium oxalate stones | Restored Ruminococcaceae_UCG-014 & Parasutterella; ↑ intestinal barrier & oxalate transporters; ↓ urinary oxalate & renal crystals | Extends FMT application to nephrolithiasis prevention; indirect relevance to CKD | An et al., 2025a |
| Healthy donor FMT | Rat | Calcium oxalate stones | Activated Muribaculaceae-Lactobacillus-Bifidobacterium network; ↓ urinary oxalate, calcium, uric acid, creatinine, urea; ↓ serum uric acid & BUN/creatinine ratio | Comprehensive metabolic improvement; potential for CKD with hyperuricemia | Wang et al., 2023c |
| FMT (MDSC-mediated) | Mouse | UUO (renal fibrosis model) | Shifted MDSCs toward monocytic phenotype; curbed inflammation & fibrosis; adoptive MDSC transfer replicated protection | Identifies MDSCs as key FMT mediators; mechanistic insight for fibrosis | Wang et al., 2025a |
| Amoxicillin-induced FMT | Mouse | Post-AKI recovery | Expanded Alistipes/Odoribacter/Stomatobaculum; ↑ Foxp3⁺CD8⁺ T cells; ↓ post-ischemic fibrosis; accelerated AKI recovery | Indirect evidence for FMT in kidney injury; not equivalent to CKD | Gharaie et al., 2023 |
| Hyperuricemic rat FMT | Rat | Post-ischemic renal injury | Transferred dysbiosis & toxic metabolites; amplified renal injury & fibrosis via NLRP3 inflammasome activation | Demonstrates causal link between dysbiotic microbiota and renal injury | Zhou et al., 2024 |
| Healthy donor FMT | Humanized mouse | IgA nephropathy | ↓ Albuminuria; restored CD89 expression; ↓ renal KC chemokine; reduced galactose-deficient IgA1 | FMT modulates IgAN severity; subtype-specific mechanism | Lauriero et al., 2021 |

Abbreviations: AhR, aryl hydrocarbon receptor; AKI, acute kidney injury; BUN, blood urea nitrogen; CKD, chronic kidney disease; CRF, chronic renal failure; DKD, diabetic kidney disease; FMT, fecal microbiota transplantation; GI, gastrointestinal; IgAN, IgA nephropathy; IL, interleukin; IS, indoxyl sulfate; LPS, lipopolysaccharide; MDSC, myeloid-derived suppressor cell; OMVs, outer-membrane vesicles; pCS, p-cresyl sulfate; SCFA, short-chain fatty acid; sIgA, secretory immunoglobulin A; TCM, traditional Chinese medicine; TNF-α, tumor necrosis factor-alpha; UT, uremic toxin; UUO, unilateral ureteral obstruction; ZO-1, zonula occludens-1.
